# Mismatch Repair Genes *Mlh1* and *Mlh3* Modify CAG Instability in Huntington's Disease Mice: Genome-Wide and Candidate Approaches

**DOI:** 10.1371/journal.pgen.1003930

**Published:** 2013-10-31

**Authors:** Ricardo Mouro Pinto, Ella Dragileva, Andrew Kirby, Alejandro Lloret, Edith Lopez, Jason St. Claire, Gagan B. Panigrahi, Caixia Hou, Kim Holloway, Tammy Gillis, Jolene R. Guide, Paula E. Cohen, Guo-Min Li, Christopher E. Pearson, Mark J. Daly, Vanessa C. Wheeler

**Affiliations:** 1 Center for Human Genetic Research, Massachusetts General Hospital, Boston, Massachusetts, United States of America; 2 Analytic and Translational Genetics Unit, Massachusetts General Hospital, Boston, Massachusetts, United States of America; 3 Program in Medical and Population Genetics, Broad Institute of MIT and Harvard, Cambridge, Massachusetts, United States of America; 4 Program of Genetics and Genome Biology, The Hospital for Sick Children, Toronto, Canada; 5 Department of Molecular Genetics, University of Toronto, Toronto, Canada; 6 Graduate Center for Toxicology and Markey Cancer Center, University of Kentucky College of Medicine, Lexington, Kentucky, United States of America; 7 Department of Biomedical Sciences, Cornell University, Ithaca, New York, United States of America; University of Virginia, United States of America

## Abstract

The Huntington's disease gene (*HTT*) CAG repeat mutation undergoes somatic expansion that correlates with pathogenesis. Modifiers of somatic expansion may therefore provide routes for therapies targeting the underlying mutation, an approach that is likely applicable to other trinucleotide repeat diseases. Huntington's disease *Hdh^Q111^* mice exhibit higher levels of somatic *HTT* CAG expansion on a C57BL/6 genetic background (B6.*Hdh^Q111^*) than on a 129 background (129.*Hdh^Q111^*). Linkage mapping in (B6x129).*Hdh^Q111^* F2 intercross animals identified a single quantitative trait locus underlying the strain-specific difference in expansion in the striatum, implicating mismatch repair (MMR) gene *Mlh1* as the most likely candidate modifier. Crossing B6.*Hdh^Q111^* mice onto an *Mlh1* null background demonstrated that *Mlh1* is essential for somatic CAG expansions and that it is an enhancer of nuclear huntingtin accumulation in striatal neurons. *Hdh^Q111^* somatic expansion was also abolished in mice deficient in the *Mlh3* gene, implicating MutLγ (MLH1–MLH3) complex as a key driver of somatic expansion. Strikingly, *Mlh1* and *Mlh3* genes encoding MMR effector proteins were as critical to somatic expansion as *Msh2* and *Msh3* genes encoding DNA mismatch recognition complex MutSβ (MSH2–MSH3). The *Mlh1* locus is highly polymorphic between B6 and 129 strains. While we were unable to detect any difference in base-base mismatch or short slipped-repeat repair activity between B6 and 129 MLH1 variants, repair efficiency was MLH1 dose-dependent. MLH1 mRNA and protein levels were significantly decreased in 129 mice compared to B6 mice, consistent with a dose-sensitive MLH1-dependent DNA repair mechanism underlying the somatic expansion difference between these strains. Together, these data identify *Mlh1* and *Mlh3* as novel critical genetic modifiers of *HTT* CAG instability, point to *Mlh1* genetic variation as the likely source of the instability difference in B6 and 129 strains and suggest that MLH1 protein levels play an important role in driving of the efficiency of somatic expansions.

## Author Summary

The expansion of a CAG repeat underlies Huntington's disease (HD), with longer CAG tracts giving rise to earlier onset and more severe disease. In individuals harboring a CAG expansion the repeat undergoes further somatic expansion over time, particularly in brain cells most susceptible to disease pathogenesis. Preventing this repeat lengthening may delay disease onset and/or slow progression. We are using mouse models of HD to identify the factors that modify the somatic expansion of the HD CAG repeat, as these may provide novel targets for therapeutic intervention. To identify genetic modifiers of somatic expansion in HD mouse models we have used both an unbiased genetic mapping approach in inbred mouse strains that exhibit different levels of somatic expansion, as well as targeted gene knockout approaches. Our results demonstrate that: 1) *Mlh1* and *Mlh3* genes, encoding components of the DNA mismatch repair pathway, are critical for somatic CAG expansion; 2) in the absence of somatic expansion the pathogenic process in the mouse is slowed; 3) MLH1 protein levels are likely to be a driver of the efficiency of somatic expansion. Together, our data provide new insight into the factors underlying the process of somatic expansion of the HD CAG repeat.

## Introduction

Huntington's disease (HD) is a fatal, dominantly inherited neurodegenerative disease, which is caused by the expansion of a CAG repeat within exon 1 of the *HTT* gene, resulting in an extended glutamine tract in the huntingtin protein (HTT) [1]. The length of the longer CAG repeat tract is the primary determinant of age of disease onset [2]. However, precise disease expression and timing are clearly modifiable by other factors, with strong evidence supporting the contribution of genetic factors [3], [4]. The identification of such factors could lead to the development of novel therapeutic interventions that modify the nature and/or pace of the HD-associated pathogenic process, and is being pursued via a number of candidate and global genetic approaches [5]. The expanded *HTT* CAG repeat is highly unstable both in the germline and in somatic tissues [6]–[13]. In somatic tissues instability is expansion-biased and prevalent in brain regions that are most susceptible to neurodegeneration [7]. Approximately 10% of expanded *HTT* CAG alleles are further expanded by at least 10 repeats in human HD postmortem brain, with dramatic increases of up to 1,000 repeats also occurring, albeit at a lower frequency [7], [11]. Given the strong CAG length-dependence of disease onset and severity, somatic expansion is predicted to accelerate the disease process. Mathematical modeling has suggested a mechanism by which somatic expansion beyond a threshold repeat length is required for clinical onset [14]. Whether in fact somatic expansion beyond a typically inherited repeat length of 40–50 CAGs is required for disease onset is unclear. Nevertheless the hypothesis that somatic expansion is at least a disease modifier is supported by the finding that longer somatic *HTT* CAG expansions are associated with an earlier residual disease onset (onset unexplained by inherited CAG length) in HD patients [11]. These data suggest that factors that modify somatic instability will also modify disease and could be targeted to delay onset or progression of HD.

Identification of modifier genes in the mouse has the potential to provide insight into disease pathways at the earliest stages of the pathogenic process. To study mechanisms of *HTT* CAG instability and pathogenesis in the mouse we have developed a series of accurate genetic Huntington's disease homologue (*Hdh* or *Htt*) CAG knock-in mice [15]–[17] that provide powerful tools to uncover genetic modifiers of early dominant, *HTT* CAG length-dependent events. Using candidate gene knockout approaches we have found that *Msh2* and *Msh3* genes, encoding a key mismatch recognition complex designated MutSβ (MSH2–MSH3 heterodimer), are essential for somatic *HTT* CAG expansion in *Hdh^Q111^* knock-in mice [18]–[20]. Similar studies using various mouse models of HD and other trinucleotide repeat diseases support a central role for the mismatch repair (MMR) pathway in somatic instability [21]–[28]. While the effects of MMR proteins on instability can vary according to the repeat sequence and its context [21]–[28], it is notable that *Msh2* and *Msh3* enhance CAG/CTG expansion both in HD and DM1 mouse models [18]–[23], [25]–[27], and *Pms2*, encoding a subunit of the MutLα (MLH1-PMS2) complex that acts downstream of mismatch recognition by MutSα (MSH2–MSH6 heterodimer) or MutSβ, was identified as a genetic enhancer of CTG expansion in a DM1 mouse model [24]. These observations highlight underlying similarities of the CAG/CTG expansion process across disease loci. Importantly, in *Hdh^Q111^* mice *Msh2* and *Msh3* promote *HTT* CAG-dependent mutant huntingtin diffuse nuclear localization and nuclear inclusion phenotypes. While the relationship between instability and nuclear huntingtin localization/inclusion phenotypes is correlative, these data support the hypothesis that somatic expansions contribute to an ongoing *HTT* CAG-dependent process [18]–[20].

An alternative approach for identifying modifiers in the mouse is to take advantage of naturally occurring strain-specific phenotypic variation. Interestingly, mouse strain-specific differences in trinucleotide repeat instability [17], [22], [29] and various HD mouse model phenotypes [17], [30], [31] have been identified. Notably, strain-specific differences in the instability of the *HTT* CAG repeat in R6/1 transgenic mice were recently found to be associated with polymorphisms in the *Msh3* gene [29]. With the aim of performing unbiased genetic screens for *HTT* CAG-dependent phenotypes we have generated congenic *Hdh^Q111^* mice on several different genetic backgrounds [17]. In a comparison of congenic B6.*Hdh^Q111^*, FVB.*Hdh^Q111^* and 129.*Hdh^Q111^* strains we previously showed that intergenerational *HTT* CAG instability, somatic *HTT* CAG instability, diffusely immunostaining nuclear huntingtin and intranuclear inclusions in striatal neurons were modified by genetic background [17], providing the opportunity to perform unbiased searches for genetic modifiers of *HTT* CAG-dependent events. Here, we set out to perform a genetic linkage study with the aim of mapping genetic modifier(s) of somatic *HTT* CAG instability in *Hdh^Q111^* mice, in order to gain further insight into factors underlying somatic instability with the potential to uncover novel targets for slowing somatic instability and/or early events in the HD pathogenic process.

## Results

### Quantification of somatic instability in congenic *Hdh^Q111^* mice

**Figure 1:**
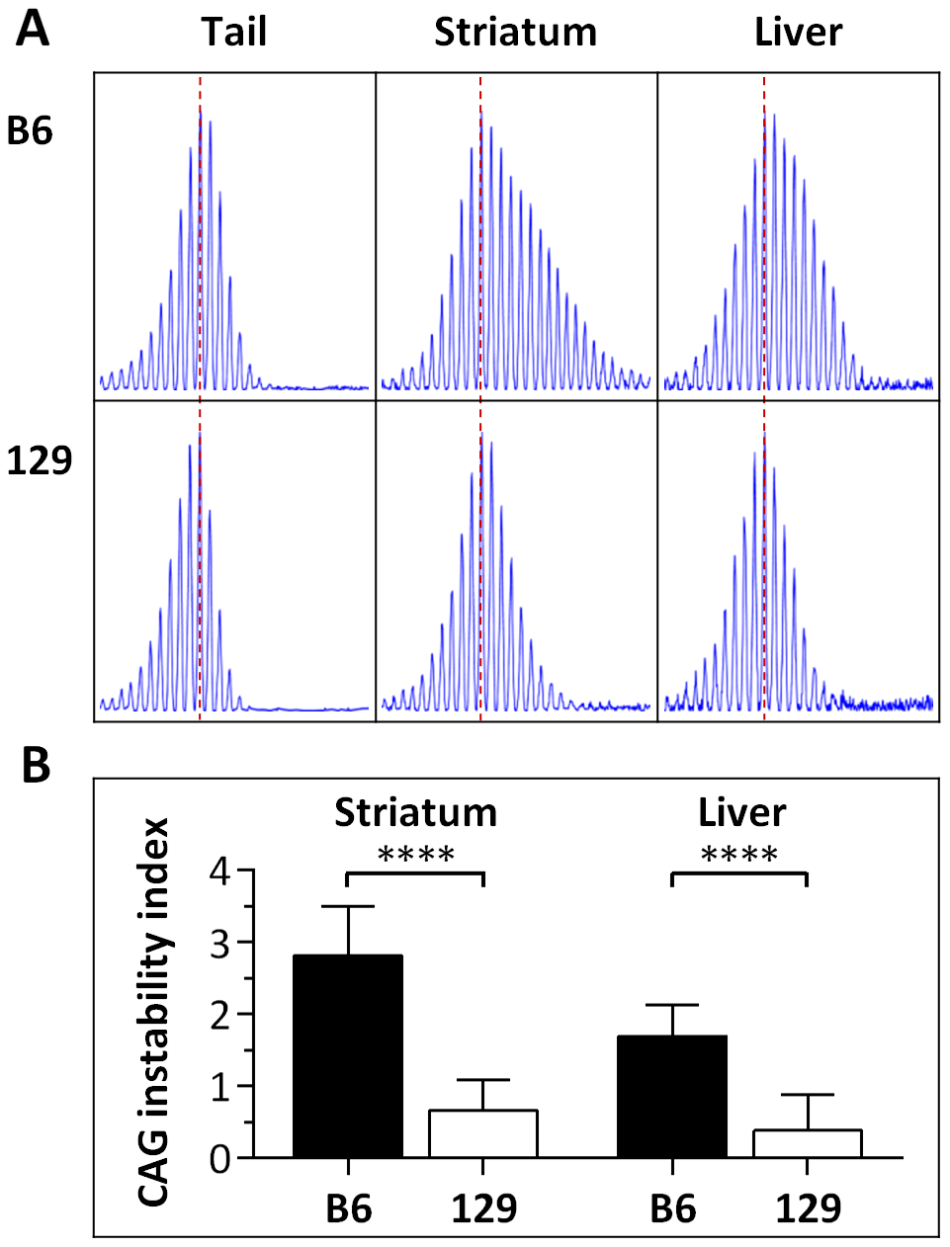
Somatic *HTT* CAG instability differs between B6.*Hdh^Q111^* ^/+^ and 129.*Hdh^Q111^* ^/+^ mice. (A) Representative GeneMapper profiles of *HTT* CAG repeat size distributions in the tail, striatum and liver of 10-week-old B6.*Hdh^Q111/+^* and 129.*Hdh^Q111/+^* mice, highlighting the altered contribution of B6 and 129 genetic background to somatic *HTT* CAG repeat expansion, as previously described [17]. Tail and striatum: B6.*Hdh^Q111/+^*, CAG116; 129.*Hdh^Q111/+^*, CAG112. Liver: B6.*Hdh^Q111/+^*, CAG113; 129.*Hdh^Q111/+^*, CAG111 (B) Quantification of CAG instability index reveals a statistically significant decrease in somatic *HTT* CAG instability in the striatum and liver of 129.*Hdh^Q111^* ^/+^ mice compared to B6.*Hdh^Q111^* ^/+^ mice. B6.*Hdh^Q111/+^* striatum, *n* = 10, CAG116.9±1.2SD; B6.*Hdh^Q111/+^* liver, *n* = 10, CAG114.3±1.2SD; 129.*Hdh^Q111/+^* striatum, *n* = 12, CAG110.9±1.2SD; 129.*Hdh^Q111/+^* liver, *n* = 9, CAG109.5±1.4SD; Bar graphs represent mean ±SD; ****, *p*<0.0001.

**Figure 2:**
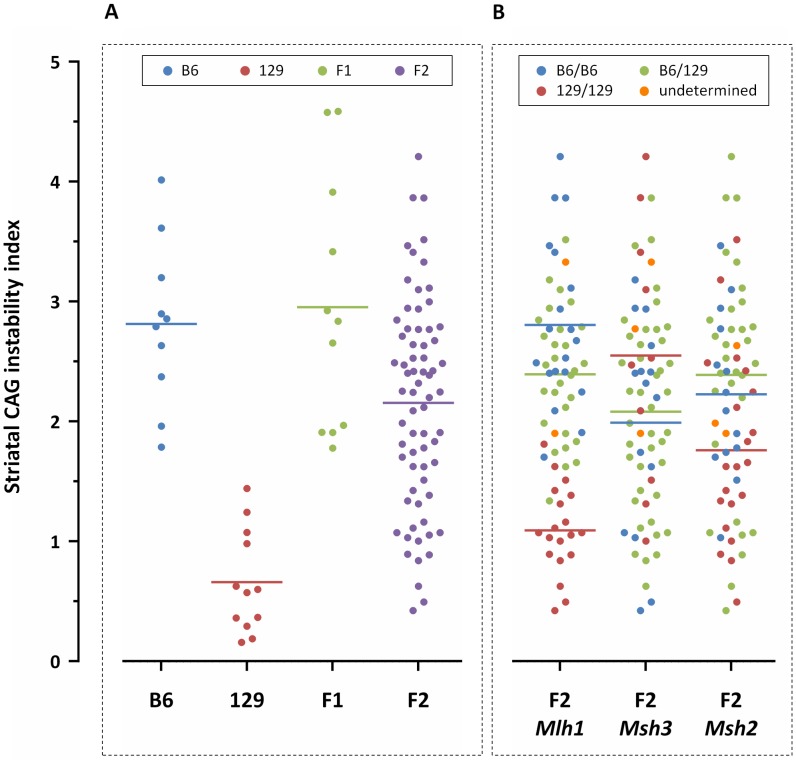
Striatal *HTT* CAG instability in 10-week-old *Hdh^Q111/+^* mice on different genetic backgrounds. Graphical representation of striatal CAG instability indices from individual (A) B6, 129, (B6x129).F1 and (B6x129).F2 mice, color-coded based on strain genetic background; and from (B) (B6x129).F2 mice color-coded by genotype at the *Mlh1*, *Msh3* and *Msh2* genes (“undetermined” indicates failed genotype). F2 mice homozygous or heterozygous for B6 *Mlh1* alleles display significantly higher levels of striatal somatic CAG instability than F2 mice homozygous for 129 *Mlh1* alleles (*p*<0.0001 for both). No relationship could be established between *Msh3* or *Msh2* genotype and striatal CAG instability. B6.*Hdh^Q111/+^*, *n* = 10, CAG116.9±1.2SD; 129.*Hdh^Q111/+^*, *n* = 12, CAG110.9±1.2SD; (B6x129).*Hdh^Q111/+^* F1, *n* = 11, CAG114.7±6.4SD; (B6x129).*Hdh^Q111/+^* F2, *n* = 69, CAG107.7±3.2SD. dbSNP markers located within MMR genes: *Mlh1*, rs30131926 and rs30174694 (concordant genotypes detected with both markers); *Msh3*, rs29551174; *Msh2*, rs33609112 and rs49012398 (concordant genotypes detected with both markers). Horizontal bars represent the mean CAG instability indices of the respective groups.

Our previous qualitative analyses revealed high and low levels of *HTT* CAG instability in striata from B6.*Hdh^Q111/+^* and 129.*Hdh^Q111/+^* mice, respectively, at both 10 and 20 weeks of age [17]. At 10 weeks of age B6.*Hdh^Q111^* ^/+^ striata display a broadened and expansion-biased CAG length distribution, in contrast to 129.*Hdh^Q111^* ^/+^ mice that display very low levels of somatic expansion (Figure 1A and [17]). By 20 weeks of age a bimodal CAG length distribution is apparent in B6.*Hdh^Q111^* ^/+^ striata, while 129.*Hdh^Q111^* ^/+^ show a broadened CAG distribution similar to that in B6.*Hdh^Q111^* striata at 10 weeks of age (Figure S1 and [17]). We were interested in identifying early-acting modifiers of instability, and therefore we determined whether the difference in instability in B6 and 129 strains at 10 weeks of age could be captured as a quantitative trait for genetic mapping experiments. We thus quantified a somatic “instability index” from GeneMapper traces of PCR-amplified *HTT* CAG repeats from B6.*Hdh^Q111/+^* and 129.*Hdh^Q111/+^* striata using a previously described method [32]. In addition, given the observation of high levels of *HTT* CAG instability in the liver of CD1.*Hdh^Q111/+^* mice [33], we also quantified instability indices in B6.*Hdh^Q111/+^* and 129.*Hdh^Q111/+^* livers. In concordance with our previous qualitative assessment [17], the quantification of instability in striatum and liver of 10-week-old mice revealed significantly higher levels in B6.*Hdh^Q111/+^* mice compared to 129.*Hdh^Q111/+^* mice (2-tailed unpaired *t*-test: *p*<0.0001 for both striatum and liver; Figure 1B). Note that there was a significant difference in the constitutive CAG repeat size between these B6 and 129 mice (2-tailed unpaired *t*-test: *p*<0.0001; Figure S2). While CAG length could, in principle, account for at least some of the difference in instability between strains, our previous analyses demonstrated a strain-specific difference in instability that was unaccounted for by CAG size alone [17], strongly indicating that identification of additional instability modifiers would be plausible. Striatal instability indices from the two strains were quite distinct (Figure 1B and Figure 2A), indicating that the instability index was likely to provide a sensitive quantitative trait for mapping genetic modifiers. Liver instability indices were less well separated between the two strains (Figure 1B), predicting less power in the ability to identify genetic modifiers of liver instability than striatal instability.

### Identification of a quantitative trait locus associated with somatic *HTT* CAG instability

**Figure 3:**
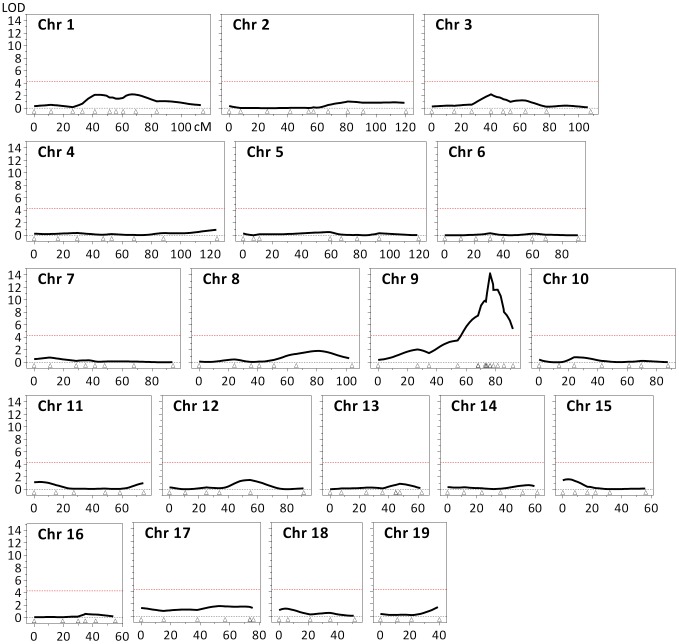
Identification of a quantitative trait locus (QTL) associated with striatal *HTT* CAG instability. Linkage analysis in 10-week-old (B6x129).*Hdh^Q111/+^* F2 mice (*n* = 69) identified a single QTL on chromosome 9, with a maximum LOD score of approximately 14 and a 2-LOD-dropoff interval of 5 Mb (chr9:107,982,655–113,057,967; GRCm38/mm10) (Figure S6). Note that the 2 markers positioned within the *Mlh1* gene (dbSNP rs30131926 and rs30174694) define the QTL peak. The red dashed line represents the threshold (LOD = 4.3) considered for the identification of significant QTLs [85]. The coordinates (cM) of the 147 genetic markers used are represented by open triangles.

Based on the findings above we used striatal instability index, which showed very good separation between B6 and 129 strains, as a quantitative phenotype for linkage mapping. Analyses of *HTT* CAG instability in striata from (B6x129).*Hdh^Q111/+^* F1 mice showed comparable instability indices to those in B6.*Hdh^Q111/+^* mice (2-tailed unpaired *t*-test: *p* = 0.11), and significantly higher instability indices than in 129.*Hdh^Q111/+^* mice (2-tailed unpaired *t*-test: *p*<0.0001) (Figure 2A), suggesting the presence of a B6 genetic locus or loci that dominantly enhance *HTT* CAG expansion. While these data were consistent with a dominant B6 modifier(s) we established an F2 intercross in order to search in an unbiased manner for both dominant and recessive modifier loci [34]. Instability indices were quantified from the striata of 69 10-week-old (B6x129).*Hdh^Q111/+^* F2 animals (Figure 2A). We observed no correlation between constitutive CAG size and striatal CAG instability in the F2 intercross mice (Pearson correlation: *R* ^2^ = 0.011, *p* = 0.39), implying the contribution of other genetic factors to the difference in *HTT* CAG instability between the two strains. Note that the genetic background of the region surrounding the *Hdh^Q111^* allele in both strains is 129 due to the etiology of the targeted ES cells, ruling out the possibility of identifying *cis*-acting modifiers. The F2 intercross mice were genotyped using an initial panel of 117 SNPs that distinguishes B6 and 129 strains (Figure S3 and Table S1). Linkage analysis identified a single quantitative trait locus (QTL) on chromosome 9 associated with striatal *HTT* CAG instability with a peak LOD score of approximately 11 (Figure S4). Notably, the MMR gene *Mlh1* is located within this interval (Figure S5). As MMR genes *Msh2* and *Msh3* had been previously established as modifiers of somatic CAG repeat expansion in *Hdh^Q111^* mice [18]–[20], additional members of this pathway would be strongly indicated as potential modifiers. In an attempt to primarily enhance resolution at this QTL, but also to specifically investigate the *Mlh1* gene, we genotyped the F2 animals for 10 additional markers distributed across the QTL region, including two markers located within the *Mlh1* gene (Figure S3 and Table S1). We also genotyped additional markers to improve overall genome coverage and specifically the coverage of the *Msh2* and *Msh3* genes. Subsequent linkage analysis that included these additional markers (total 147 SNPs) not only confirmed the mapping of a single QTL on chromosome 9 (Figure 3), but also significantly narrowed down the implicated genomic region to an interval of approximately 5 Mb (chr9:107,982,655–113,057,967; GRCm38/mm10) (Figure S6). This genomic region, which represents a 95% confidence interval, is defined by the markers encompassing a 2-LOD drop-off from the peak LOD score [35]. Interestingly, the markers at the *Mlh1* locus defined the QTL peak, which was significantly increased to a LOD score of approximately 14 (Figure 3 and Figure S6). We did not find any evidence for linkage to the *Msh2* or *Msh3* genes on chromosomes 17 and 13, respectively (Figure 2B and Figure 3). Note that constitutive CAG repeat lengths in the F2 mice did not cluster with genotype at the *Mlh1* locus (Figure S2), consistent with the lack of correlation between constitutive CAG length and instability index in these mice. The chromosome 9 QTL explains approximately 60% of the variance in striatal instability, with the remaining 40% of the variance being attributable to differences within the parental strains, strongly supporting this locus as the single major modifier of instability between the two strains. Further, the effect of the QTL was consistent with the B6 allele acting in a dominant fashion (Figure 2).

In addition to *Mlh1*, the implicated genomic region contains numerous genes (Figure S6), none of which we are able to objectively exclude as a modifier based on our genetic data. However, none of these genes has been shown or is suspected to be involved in repeat instability. Past observations that the MMR pathway plays a major role in modulating somatic *HTT* CAG instability, together with the highest LOD scores observed with two markers that were located within the *Mlh1* gene, strongly suggest that this MMR gene is a likely candidate modifier underlying the chromosome 9 QTL.

### *Mlh1* is a modifier of somatic *HTT* CAG instability and nuclear mutant huntingtin

**Figure 4:**
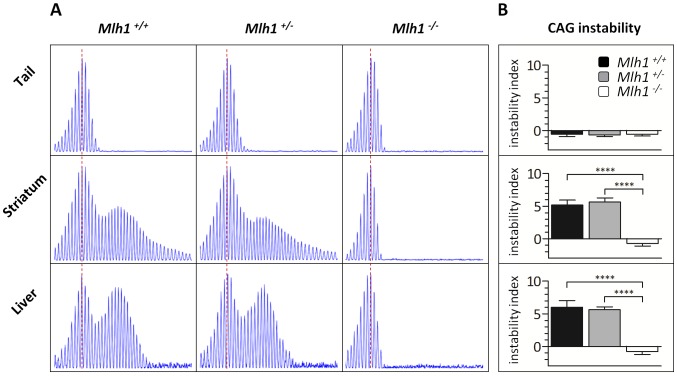
*Mlh1* is required for striatal and liver *HTT* CAG repeat instability in B6.*Hdh^Q111/+^* mice. (A) Representative GeneMapper profiles of *HTT* CAG repeat size distributions in the tail, striatum and liver of 22-week-old B6.*Hdh^Q111/+^* mice on different *Mlh1* genetic backgrounds. *Mlh1^+/+^*, CAG113; *Mlh1^+/−^*, CAG113; *Mlh1^−/−^*, CAG110. (B) Quantification of striatal and liver *HTT* CAG instability indices in these mice reveals a statistically significant decrease in *HTT* CAG instability in the absence of *Mlh1*. *Mlh1^+/+^*, CAG115.3±4.9SD, *n* = 6; *Mlh1^+/−^*, CAG112.0±2.1SD, *n* = 6; *Mlh1^−/−^*, CAG109.3±2.6SD, *n* = 6. Bar graphs represent mean ±SD. ****, *p*<0.0001.

Based on our above findings we hypothesized that *Mlh1* was a modifier of somatic *HTT* CAG expansion. Therefore, to investigate the role of the *Mlh1* gene in somatic *HTT* CAG expansion we crossed B6.*Hdh^Q111^* and *Mlh1* null mice (B6) [36] and quantified CAG repeat size distributions in tail, striatum and liver of 22-week-old B6.*Hdh^Q111/+^* animals on *Mlh1^+/+^*, *Mlh1^+/−^* and *Mlh1^−/−^* genetic backgrounds (Figure 4). By 22 weeks a bimodal repeat size distribution was apparent both in striata and liver of *Mlh1^+/+^* mice, as previously shown [33]. *Mlh1* ^+/−^ mice exhibited similar levels of instability in striatum and liver to those in *Mlh1* ^+/+^ mice (2-tailed unpaired *t*-tests: striatum, *p* = 0.30; liver, *p* = 0.47). However, no instability was present in either of these tissues in *Mlh1^−/−^* mice (2-tailed unpaired *t*-test: *p*<0.0001 compared to *Mlh1* ^+/+^). These findings demonstrate that *Mlh1* is absolutely required for somatic *HTT* CAG expansions in B6.*Hdh^Q111^* mice, and provide compelling evidence that genetic differences between B6 and 129 strains at the *Mlh1* gene are likely to underlie the difference in somatic instability between these two strains. Note that the effect of the *Mlh1* knockout is to eliminate somatic *HTT* expansion at 22 weeks of age, while the 129 genetic background results in reduced somatic expansion at the same age (Figure S1). Therefore, if *Mlh1* genetic variants do indeed underlie the difference in striatal instability between B6 and 129 strains, such variants are likely to confer a moderate effect on MLH1.

**Figure 5:**
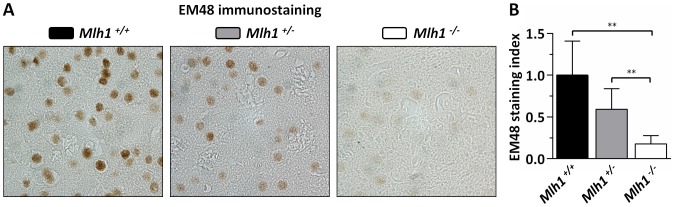
*Mlh1* is an enhancer of nuclear mutant huntingtin immunostaining in B6.*Hdh^Q111/+^* mice. (A) Representative EM48 immunostained histological sections from striata of 22-week-old B6.*Hdh^Q111/+^* mice on different *Mlh1* genetic backgrounds. *Mlh1^+/+^*, CAG113; *Mlh1^+/−^*, CAG108; *Mlh1^−/−^*, CAG110. (B) Quantification of diffuse nuclear EM48 staining demonstrates a statistically significant reduction in the absence of *Mlh1*. *Mlh1^+/+^*, CAG115.3±4.9SD, *n* = 6; *Mlh1^+/−^*, CAG112.0±2.1SD, *n* = 6; *Mlh1^−/−^*, CAG109.2±2.9SD, *n* = 5. Bar graphs represent mean ±SD. **, *p*<0.01.

We have previously shown that deletion of mismatch repair genes *Msh2* or *Msh3* is sufficient to delay the accumulation/epitope accessibility of diffusely immunostained mutant huntingtin in the nuclei of striatal neurons [18]–[20]. This early phenotype, which is both dominant and CAG length-dependent [16], is a sensitive marker of the ongoing pathogenic process in these mice. To determine whether *Mlh1* also modified this phenotype we quantified diffusely-immunostained nuclear huntingtin in striatal sections of 22-week-old B6.*Hdh^Q111/+^* animals on *Mlh1^+/+^*, *Mlh1^+/−^* and *Mlh1^−/−^* genetic backgrounds (Figure 5). Nuclear huntingtin immunostaining intensity was reduced in *Mlh1^+/−^* striata to approximately 60% of *Mlh1^+/+^* levels, although this difference did not reach statistical significance (2-tailed unpaired *t*-test: *p* = 0.06). In *Mlh1^−/−^* striata nuclear huntingtin immunostaining intensity was dramatically reduced to approximately 18% of *Mlh1^+/+^* levels (2-tailed unpaired *t*-test: *p* = 0.0018). Together, these findings reveal *Mlh1* as a genetic enhancer both of somatic expansion and of an early CAG length-dependent phenotype in B6.*Hdh^Q111/+^* mice, supporting the hypothesis that somatic expansion accelerates *HTT* CAG-dependent events.

### *Mlh3* is a modifier of somatic *HTT* CAG repeat instability

**Figure 6:**
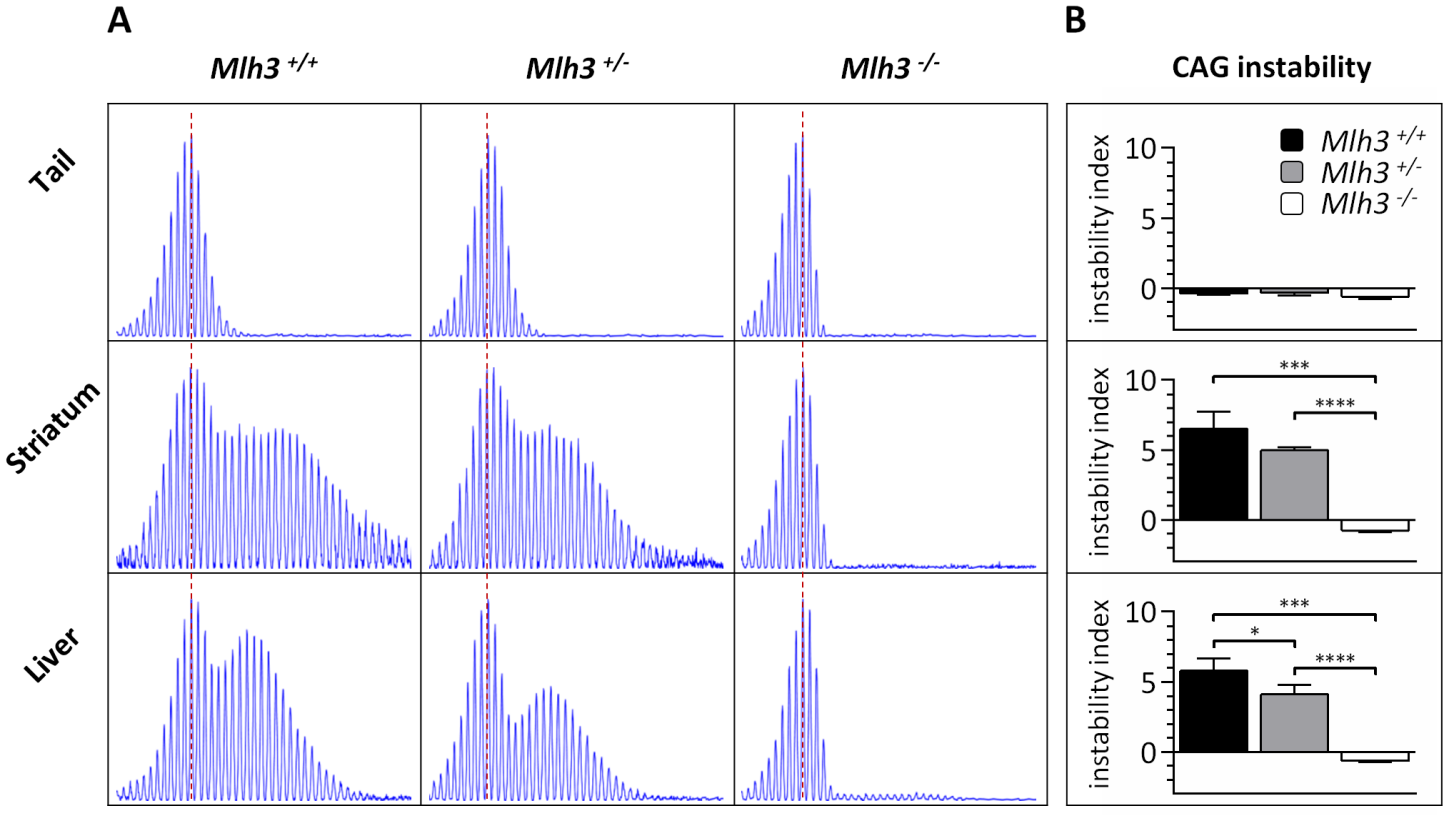
*Mlh3* is required for striatal and liver *HTT* CAG repeat instability in B6.*Hdh^Q111/+^* mice. (A) Representative GeneMapper profiles of *HTT* CAG repeat size distributions in the tail, striatum and liver of 24-week-old B6.*Hdh^Q111/+^* mice on different *Mlh3* genetic backgrounds. *Mlh3^+/+^*, CAG103; *Mlh3^+/−^*, CAG101; *Mlh3^−/−^*, CAG102. (B) Quantification of striatal and liver *HTT* CAG instability indices in these animals reveals a statistically significant suppression of *HTT* CAG instability in the absence of *Mlh3*. *Mlh3^+/+^*, CAG103.3±1.5SD, *n* = 3; *Mlh3^+/−^*, CAG101.3±0.5SD, *n* = 4; *Mlh3^−/−^*, CAG101.3±0.6SD, *n* = 3. Bar graphs represent mean ±SD. *, *p*<0.05; ***, *p*<0.001; ****, *p*<0.0001.

Given the critical role of MLH1 in somatic *HTT* CAG expansion we were interested in investigating further this MLH1-mediated pathway. It is known that MLH1 is an obligate subunit of three MutL complexes: MutLα (MLH1-PMS2), MutLβ (MLH1-PMS1) and MutLγ (MLH1–MLH3) (reviewed in [37], [38]). These MutL heterodimers are essential downstream factors in MMR and are recruited to the MMR reaction following the binding of mismatched DNA by MutSα (MSH2–MSH6) or MutSβ (MSH2–MSH3). Outside of its role in meiotic recombination [39], MutLγ appears to function predominantly with MutSβ both in somatic and germ cells [40], [41]. Given the specific requirement for MutSβ in somatic CAG expansion in *Hdh^Q111^* mice [19] and other mouse models of CAG/CTG disease [22], [25], [26], we hypothesized that MLH3 may also play a major role in somatic expansion. A role for MLH3 had also been suggested from findings in a mouse model of myotonic dystrophy type 1 in which knockout of *Pms2*, encoding MLH1's major binding partner, reduced the rate of somatic CTG expansion by ∼50%, but did not eliminate somatic expansions [24]. We therefore crossed B6.*Hdh^Q111^* with *Mlh3* null mice (B6) [39] and quantified *HTT* CAG repeat size distributions in the tail, striatum and liver of 24-week-old B6.*Hdh^Q111/+^* animals on *Mlh3^+/+^*, *Mlh3^+/−^* and *Mlh3^−/−^* genetic backgrounds (Figure 6). Slightly reduced striatum- and liver-specific CAG instability was observed in *Mlh3^+/−^* mice when compared to *Mlh3^+/+^* animals (2-tailed unpaired *t*-tests: striatum, *p* = 0.06; liver, *p* = 0.03). Interestingly, no instability was present in *Mlh3^−/−^* striatum or liver (2 tailed unpaired *t*-tests: *p*<0.0001 for both tissues compared to *Mlh3^+/+^*), demonstrating, as for MLH1, that MLH3 is absolutely required for somatic *HTT* CAG instability in B6.*Hdh^Q111^* mice, and implying that MutLγ dimers act in this process. The slight reduction of instability in *Mlh3* ^+/−^ mice (Figure 6), not apparent in *Mlh1* ^+/−^ mice (Figure 4) suggests that *Mlh3* may be a limiting factor in somatic expansion, as previously reported for *Msh3* [19], [26]. The relatively strong impacts of heterozygous loss of *Mlh3* and *Msh3* compared to heterozygous loss of the *Mlh1* and *Msh2* genes encoding their respective binding partners may be explained in part by the lower levels of MSH3 compared to MSH2 and of MLH3 compared to MLH1 [42], [43].

### The *Mlh1* locus is highly polymorphic between B6 and 129 strains

**Figure 7:**
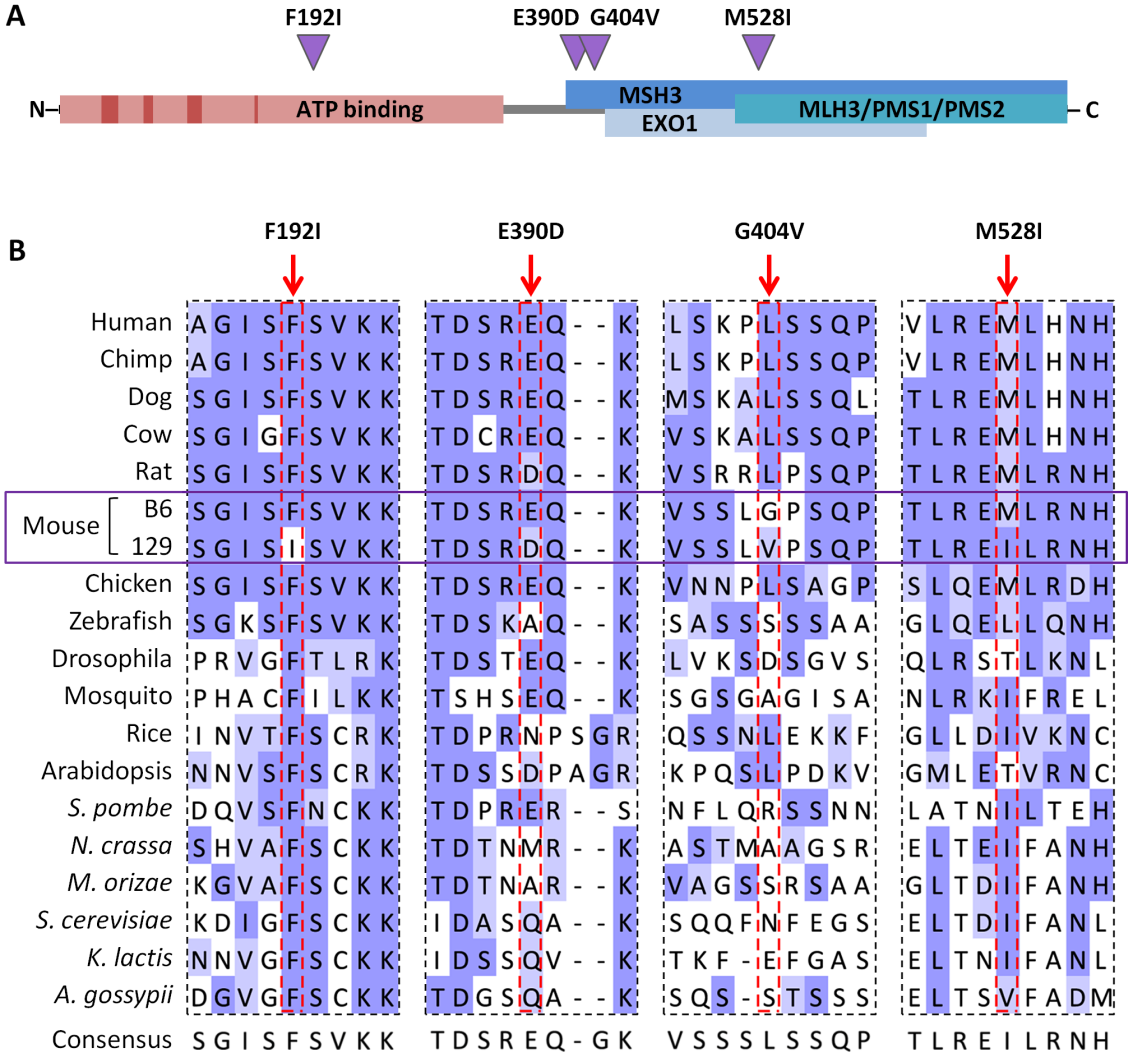
B6-129 MLH1 protein polymorphisms. (A) Schematic representation of the murine MLH1 protein showing the location of B6-129 nonsynonymous polymorphisms identified (purple triangles) and their positions relative to conserved ATP binding motifs and ATP binding domain (dark and light red boxes, respectively) [46], as well as proposed MMR protein interaction domains (blue boxes) [41]. (B) Cross-species alignment of B6 and 129 MLH1 proteins in regions encompassing the polymorphic sites between the two strains. Protein sequence alignment was performed using Clustal Omega [96] and visualized in Jalview [97] with BLOSUM62 color scheme: white, residue does not match the consensus residue at that position; light blue, residue does not match the consensus residue but the two residues have a positive BLOSUM62 score; dark blue, residue matches consensus sequence.

While our linkage peak contained many genes, given the finding that *Mlh1* is necessary for somatic *HTT* CAG expansion, we focused on this gene as the most likely candidate modifier at the linked chromosome 9 locus. We initially investigated polymorphisms at the *Mlh1* locus between C57BL/6NCrl and 129S2/SvPasCrlf strains (in which the QTL mapping was carried out) by sequencing all *Mlh1* exons as well as the immediate 5′ and 3′ flanking regions (2.6 kb and 2 kb respectively). A relatively high frequency of SNPs was identified in the 5′UTR of *Mlh1* (8 SNPs in an 84 bp region), and a single SNP was detected in the 3′UTR (Table 1). We also identified 14 exonic SNPs, 4 of which result in an amino acid change: F192I, E390D, G404V and M528I (Figure 7). A subsequent investigation of the *Mlh1* locus in the highly related C57BL/6NJ and 129S1/SvImJ strains using whole genome sequencing data from the Mouse Genomes Project [44], [45] confirmed all of the B6-129 polymorphisms initially identified by us by Sanger sequencing. It also resulted in the identification of a large number of additional polymorphims between B6 and 129 strains, dispersed throughout the entire *Mlh1* locus (Table 1 and Figure S7). In total, 642 polymorphisms were identified in a 64 kb region encompassing the *Mlh1* gene, averaging approximately 10 polymorphims per kb. In comparison to the average genome wide variation between B6 and 129 strains of 2.4 polymorphisms per kb the *Mlh1* gene exhibits a high degree of variation, with only 5.9% of the genome displaying a relative density greater than or equal to 10 polymorphism per kb (see Materials and Methods and [44]). It is noteworthy that the haplotype across this 64 kb region in FVB/N and DBA/2J strains that display similar high somatic *HTT* CAG instability to B6 strains is highly similar to the B6 haplotype (Figure S7 and Figure S8). While this finding was consistent with a B6-like haplotype at the *Mlh1* locus underlying high instability, the relatedness of the B6, FVB/N and DBA/2J haplotypes did not provide the means to further refine the putative instability-associated region(s).

**Table 1:** The *Mlh1* locus is highly polymorphic between B6 and 129 strains.

Genomic region		Number of polymorphisms
**5′ of ** ***Mlh1*** ** (16 kb)**		85
***Mlh1*** ** (43 kb)**	5′ UTR	8
	exonic	14 (4*)
	intronic	518
	3′ UTR	1
**3′ of ** ***Mlh1*** ** (5 kb)**		16
**Total (64 kb)**		642

* Nonsynonymous SNPs.

DNA polymorphisms (SNPs, short indels and structural variants) identified at a 64 kb region surrounding the *Mlh1* locus (chr9:111,223,496–111,287,496; GRCm38/mm10) as determined by Sanger sequencing (C57BL/6N vs. 129S2/SvPasCrlf) and whole genome sequencing (C57BL/6NJ vs. 129S1/SvImJ) performed as part of the Mouse Genomes Project (Wellcome Trust Sanger Institute) [44], [45]. The *Mlh1* locus is highly polymorphic between B6 and 129 strains, averaging approximately 10 polymorphisms per kb (Figure S7 and [44]).

All 4 nonsynonymous SNPs are suspected to be in key protein domains: F192I falls within the putative ATP binding domain, though outside conserved ATP binding motifs [46]; E390D and G404V are within a domain thought to be necessary for interaction with MSH3 [41], and M528I is in a region implicated in interaction with MSH3, EXOI, MLH3, PMS1 and PMS2 [41] (Figure 7A). Note that none of these variants has been identified in human *MLH1* [47]. Cross-species alignment of MLH1 proteins (Figure 7B) shows that the Phe residue at aa192 of the B6 MLH1 protein was fully conserved across the organisms investigated, with an Ile residue at this position present in 129 strains. At positions 390 and 528 the B6-like amino acid is highly conserved, mainly in higher organisms, while the 129-like amino acid at position 528 is also well represented, particularly among lower organisms. In contrast, aa404 is poorly conserved. While none of the SNP variants alters the general chemical similarity of the amino acids, the conservation data indicate that the F192I substitution may have a functional impact. This is supported by PolyPhen-2 analysis [48] predicting E390D, G404V and M528I to be “benign”, but predicting the F192I mutation to be “probably damaging” with a maximum confidence score.

### B6 and 129 MLH1 proteins do not differ in their intrinsic DNA repair capacity but repair of CAG slip-outs is MLH1 dose-dependent

**Figure 8:**
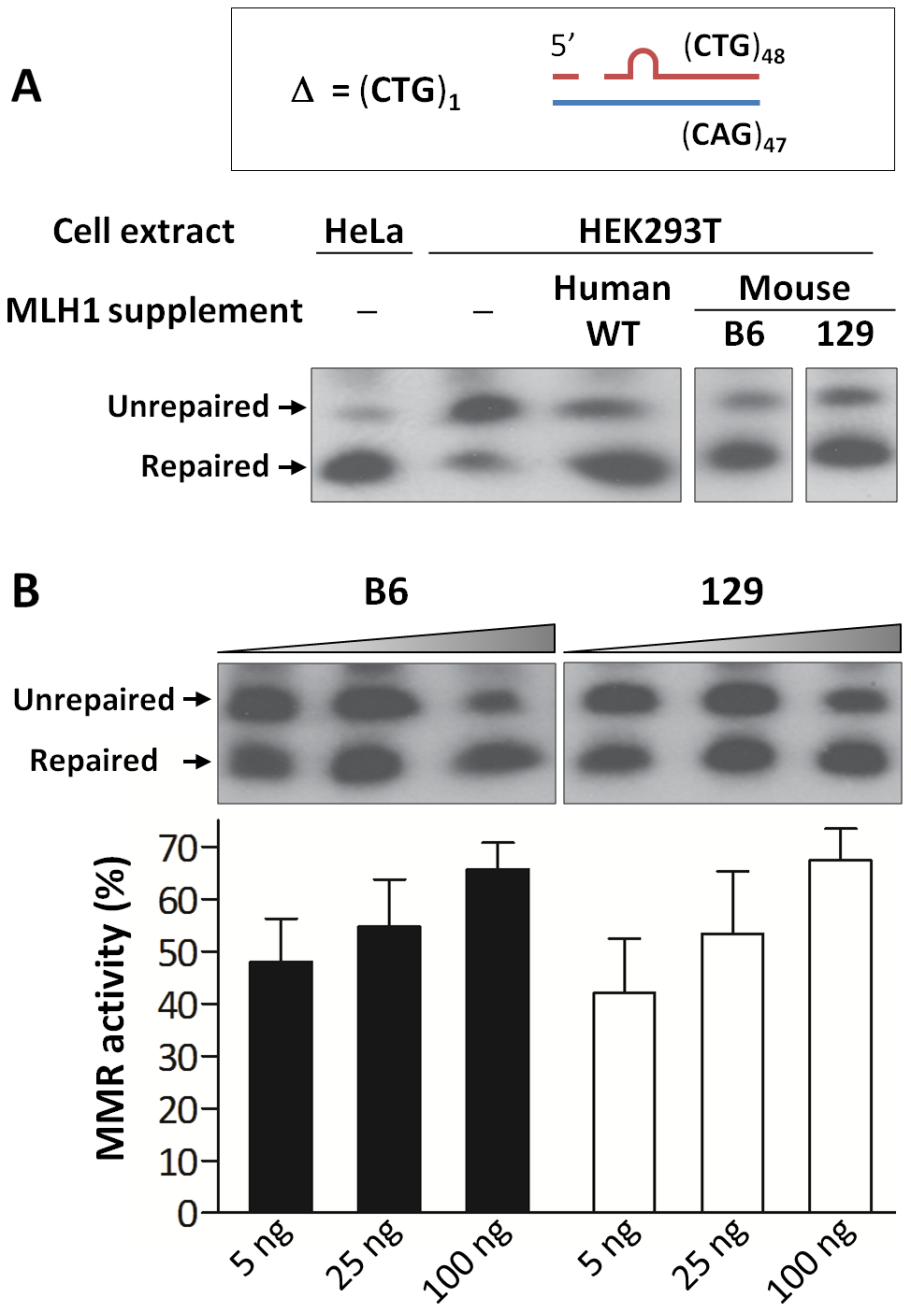
Repair of a single CTG slip-out in a cell-free MMR assay is MLH1 dose-dependent. (A) Short slipped-DNA repair using HeLa or HEK293T (MutLα-deficient) whole cell extracts complemented with equal amounts (100 ng) of purified MutLα protein complexes: hMLH1-hPMS2, mMLH1.B6-hPMS2 or mMLH1.129-hPMS2. Both B6 and 129 MLH1 proteins show ability to repair the mismatch when in a complex with hPMS2. The individual lanes represented are from the same blot. (B) Repair using MutLα-deficient HEK293T cell extracts complemented with increasing concentrations (5, 25 and 100 ng) of either mMLH1.B6-hPMS2 or mMLH1.129-hPMS2 protein complexes. Quantification of repair suggests that both B6 and 129 MLH1 proteins are comparably efficient at repairing CTG slip-outs. In addition, it suggests a MutLα dose-dependency, with higher concentrations of mMLH1-hPMS2 resulting in higher levels of MMR activity (*p* = 0.0013). The individual lanes represented are from the same blot and the experiment was reproduced three times. Bars graphs represent mean ±SD.

The highly polymorphic nature of the *Mlh1* gene indicated that delineation of the functional polymorphism(s) that drives the difference in instability between B6 and 129 mice may well be complex. However, based on the above prediction that at least the F192I substitution may have a functional impact we tested the simplest hypothesis that the B6 and 129 versions of MLH1 have different levels of activity. As there is currently no good assay for MutLγ function, we performed cell-free assays using MutLα (MLH1-PMS2 complexes), known to be required to repair G-T mismatches and single repeat slip-outs of CAG/CTG tracts [49], [50], in order to provide the most sensitive test of B6 and 129 MLH1 function. We thus cloned and co-purified B6-like (mMLH1.B6-hPMS2) and 129-like (mMLH1.129-hPMS2) MutLα proteins (Figure S11) and assessed the ability of these proteins (containing all 4 amino acid differences; Figure 6A) to repair various DNA substrates using cell-free assays. The results revealed that B6 and 129 MLH1 proteins displayed no overt difference in their abilities to repair a G-T mismatch (Figure S12). In addition, the human MLH1 protein carrying the F192I mutation showed MMR activity comparable to that of wild-type human MLH1 (Figure S12). We then tested the ability of B6 and 129 MLH1 proteins to repair a single CTG slip-out (CAG)_47_•(CTG)_48_ [50], [51], a potential intermediate in the expansion process, as requirements for processing of slipped-DNAs formed by trinucleotide repeats may more closely resemble those that ultimately result in CAG expansion in mice. As shown previously [50], complementation of MLH1- and PMS2-deficient HEK293T cells with wild-type human MutLα restored repair activity (Figure 8A). Complementation with mMLH1.B6-hPMS2 or mMLH1.129-hPMS2 MutLα complexes also restored repair to similar efficiencies (Figure 8A). Titration of concentration of the B6-like and 129-like MutLα complexes confirmed similar repair efficiencies between the MLH1 protein from the two mouse strains at each concentration (2-tailed unpaired *t*-tests: 5 ng, *p* = 0.477; 25 ng, *p* = 0.885; 100 ng, *p* = 0.736), but also demonstrated a statistically significant MutLα dose dependency of CTG slip-out repair (linear regression: *R^2^* = 0.557, p = 0.0004; Figure 8B). Together, these results demonstrate that B6 and 129 MLH1 proteins, in the context of the mixed-species MutLα complex, do not differ substantially in their G-T mismatch or CTG slip-out repair activities and that the F192I mutation in the human protein does not have a significant functional impact. This suggests that if *Mlh1* gene variations are in fact the source of the CAG repeat instability differences between the B6 and 129 mouse strains *in vivo*, this is unlikely to be due to major differences in MLH1 protein activity within the context of the MutLα complex. However, the dose-dependence of the MutLα complex in the CTG slip-out repair assay indicated that differential MLH1 protein levels between the two strains may be relevant to their different levels of instability *in vivo*.

### *Mlh1* mRNA and protein levels are reduced in 129 versus B6 mice

**Figure 9:**
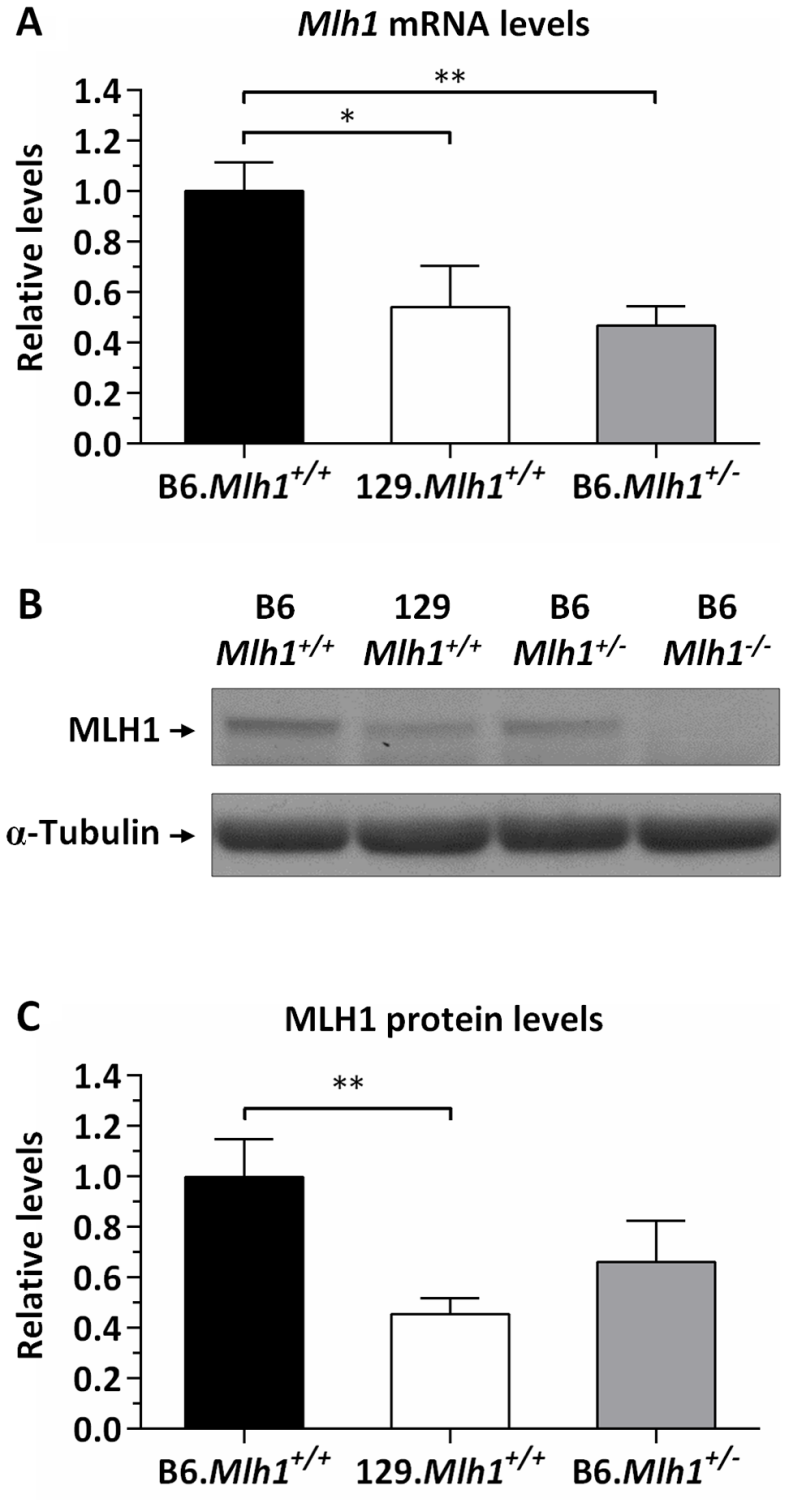
Reduced MLH1 expression in 129 versus B6 mice. Quantification of MLH1 (A) mRNA and (B, C) protein levels in the striatum of B6.*Mlh1^+/+^*, 129.*Mlh1^+/+^* and B6.*Mlh1^+/−^* 10-week-old mice (*n* = 3). (A) Striatal *Mlh1* mRNA levels (TaqMan Mm00503449_m1, exons 11–12) in 129.*Mlh1^+/+^* mice were significantly reduced by approximately 50% when compared to B6.*Mlh1^+/+^* (*p*<0.05), and were comparable to levels in B6.*Mlh1^+/−^* mice. (B, C) Western blot analysis of MLH1 protein revealed significantly reduced levels in 129.*Mlh1^+/+^* striata compared to B6.*Mlh1^+/+^* striata. Bar graphs represent mean ±SD. *, *p*<0.05; **, *p*<0.01.

The cell-free CTG slip-out repair assays suggested that levels of MLH1 may impact the ability of MutL complexes to execute a repair process that results in CAG expansion *in vivo*. We therefore assessed whether *Mlh1* expression levels differed between the B6 and 129 strains that exhibit comparatively high and low *HTT* CAG instability, respectively. Striatal *Mlh1* mRNA amount was significantly reduced in 129 mice to 54% of that in B6 mice (2-tailed unpaired *t*-test: *p* = 0.017), reaching approximately the same mRNA level as that in B6.*Mlh1* ^+/−^ mice (Figure 9A). Striatal *Mlh1* mRNA levels were consistently reduced in 129 mice across 3 distinct regions of the primary *Mlh1* transcript (exons 4–5, 11–12, and 18–19), and in various other tissues (cerebellum, liver, jejunum and ileum) to between 25% and 50% of B6 levels (Figure S13). Analysis of MLH1 protein by western blot showed similarly reduced protein levels in 129 compared to B6 striata (Figure 9B, C). In contrast to the mRNA, however, the MLH1 protein level in B6.*Mlh1* ^+/−^ mice was intermediate between that in B6 (*Mlh1* ^+/+^) and 129 striata (Figure 9B, C). We were unable to detect any evidence for novel isoforms or truncation products in the 129 mice (Figure S14).

**Figure 10:**
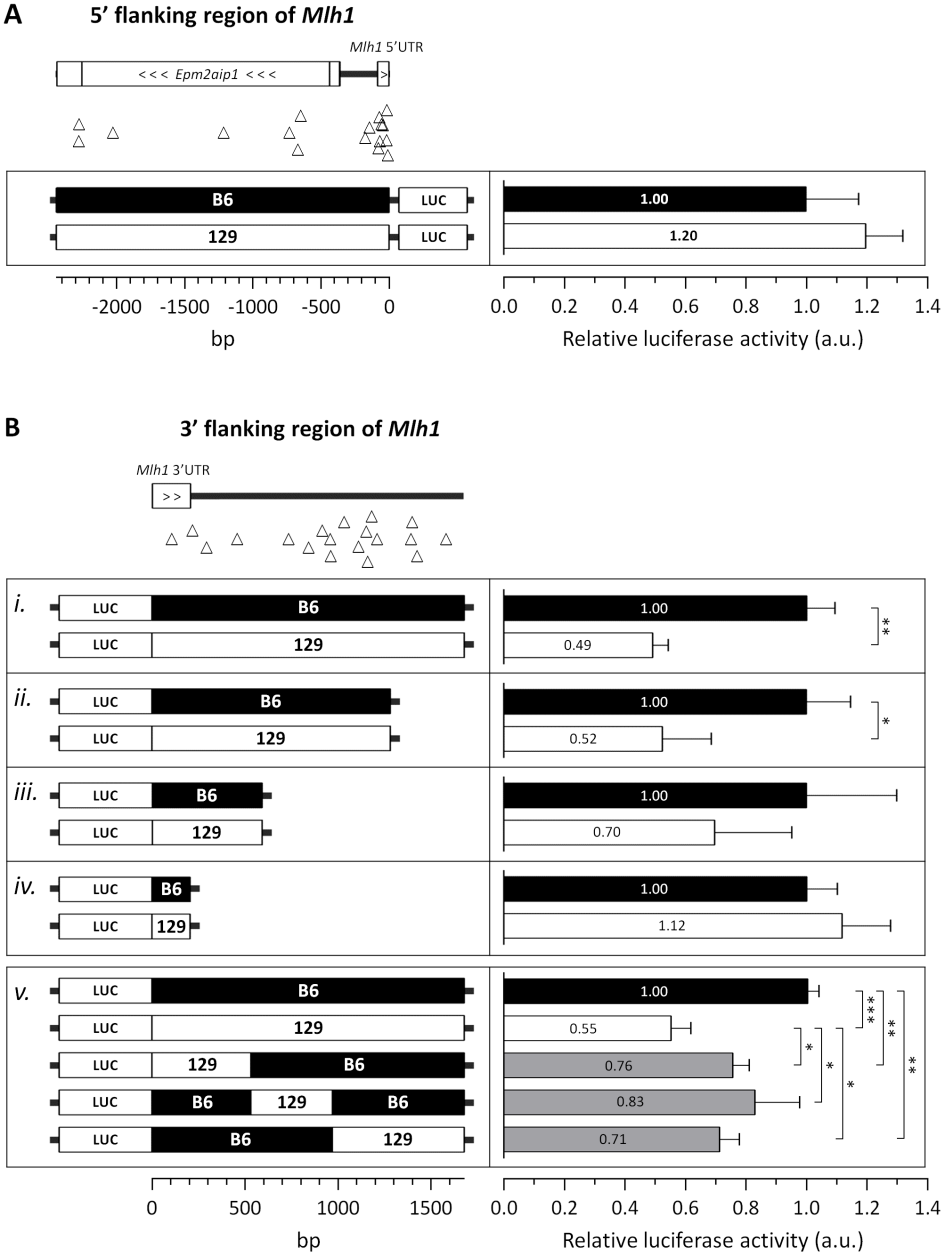
The 129 and B6 3′-flanking regions of *Mlh1* confer differential mRNA regulation. Investigation of the regulatory potential of B6 and 129 immediate (A) 5′- and (B) 3′-flanking regions of *Mlh1* using dual luciferase reporter assays. (A) The immediate 5′-flanking region of *Mlh1* containing 17 B6-129 polymorphisms (2,441 bp) was used to drive firefly luciferase expression. (B) The immediate 3′-flanking region of *Mlh1* (*i–iv*) containing either 19, 15, 4 or 1 B6-129 polymorphism(s) (1,676 bp, 1,280 bp, 591 bp and 205 bp, respectively) was cloned downstream of a firefly luciferase gene. “Swap” constructs (*v*) of the immediate 3′-flanking region of *Mlh1* containing either 4, 5 or 10 129 polymorphisms (530 bp, 438 bp and 708 bp, respectively; total 1676 bp) were cloned downstream of a firefly luciferase gene. Relative luciferase activity was determined by normalization to internal *Renilla* luminescence and determined relative to the analogous B6 construct. B6-129 polymorphisms are represented by open triangles. Bar graphs represent mean ±SD. *, *p*<0.05; **, *p*<0.01; ***, *p*<0.001.

Given the difference in *Mlh1* mRNA levels between B6 and 129 strains we investigated possible polymorphisms that might underlie this difference. As we had identified polymorphisms in both 5′ and 3′ regulatory regions of *Mlh1* (Table 1 and Figure S7) we tested whether either the immediate 5′- or 3′-flanking regions (2.4 kb and 1.7 kb, respectively) of either the B6 or 129 *Mlh1* gene were able to drive differential steady state levels of a luciferase reporter gene (Figure 10). As shown in Figure 10A there was no significant difference in firefly luciferase activity when either the B6 5′ region or the 129 5′ region was used to drive firefly luciferase expression (2-tailed unpaired *t*-test: *p* = 0.18). In contrast, when the 3′ region was cloned downstream of the firefly luciferase gene (Figure 10B, panel i), whose expression was driven from the SV40 promoter, the 129 3′ region resulted in a ∼2-fold reduction in firefly luciferase activity compared to the B6 3′ region (2-tailed unpaired *t*-test: *p* = 0.012). These results suggest that polymorphisms in this 3′ genomic region may be relevant to the ∼2-fold reduction of *Mlh1* mRNA seen *in vivo* in 129 mice compared to B6 mice (Figure 9). In an effort to narrow down the polymorphisms within this region that contributed to the differential luciferase expression we performed further luciferase reporter assays in which the 3′ genomic region from either strain was either successively deleted (Figure 10B, panels ii–iv) or in which the original 1.7 kb 3′ region from the B6 *Mlh1* gene was substituted with different subdomains of 129 genomic sequence (Figure 10B, panel v). The deletion experiments (panels ii, iii, iv) indicated that neither the single polymorphism within the 3′UTR (Figure 10B, panel iv), nor the 3′ most 4 polymorphisms (Figure 10, panel ii) contributed to the differential firefly luciferase expression. The data indicated that polymorphisms both in the 129 3′ genomic region from 205 bp to 591 bp (panel iii) and in the genomic region from 591 bp to 1,280 bp (panels ii and iii) contributed to the 2-fold reduction in firefly luciferase activity. The domain “swap” experiments (panel v) showed partial reduction of firefly luciferase activity when each of three B6 genomic regions was substituted with 129 sequence, confirming the contribution of multiple 3′ polymorphisms to the differential firefly luciferase activity.

Taken together, the results of our expression analyses indicate that genetic differences between B6 and 129 strains result in lower steady state *Mlh1* mRNA levels in 129 compared to B6 mice. Luciferase reporter assays suggest that this may, at least in part, be driven by a combination of polymorphisms 3′ to the *Mlh1* coding region. In addition, the lower relative level of MLH1 protein in 129 versus B6.*Mlh* ^+/−^ striata despite similar *Mlh1* mRNA levels (Figure 9) further suggests that genetic differences between these strains also act post-transcriptionally. While we currently have no good evidence for altered protein isoforms/truncation products in 129 versus B6 mice, the high degree of variation at the *Mlh1* locus suggests that mechanisms that might impact the levels of full-length protein in 129 mice, including altered mRNA splicing, warrant further investigation. Our data indicate, therefore, that the low *HTT* CAG instability in 129 versus B6 mice may be in part driven by reduced levels of MLH1 protein. These findings are consistent with the strong genetic linkage of an instability modifier to the *Mlh1* gene and indicate that B6 versus 129 variants may act in multiple ways to ultimately determine the different MLH1 protein levels in these strains.

## Discussion

Here we report the first unbiased QTL mapping study in a mouse model of Huntington's disease, in which we have mapped a locus that modifies the somatic expansion of the *HTT* CAG repeat. Using a quantitative measure of striatal *HTT* CAG instability we were able to detect a single modifier locus of large effect using as few as 69 F2 intercross mice. These results indicate that, depending on the number and effect size of the modifier loci, an intercross mapping strategy in congenic *Hdh^Q111^* strains is a potentially powerful approach that could be applied to identify modifiers of a variety of *HTT* CAG-dependent phenotypes.

While our genetic data do not exclude a role for other gene(s) within the linked locus as instability modifiers, the high LOD score observed with markers positioned over the *Mlh1* gene and the knowledge that this gene is essential for somatic *HTT* CAG instability provide compelling evidence that *Mlh1* is the likely genetic modifier underlying the difference in striatal *HTT* CAG instability between the B6 and 129 *Hdh^Q111^* mice. Further experiments would be needed to determine whether the same QTL contributes to the difference in liver instability between B6 and 129 strains, and/or whether other genetic loci might play a role. Two additional genes, *Trex1* and *Atrip*, located within the 2 LOD drop-off interval, are involved in DNA repair [52], [53]. However, in a comparison with two additional unstable strains, FVB.*Hdh^Q111^* and DBA.*Hdh^Q111^* (Figure S8), we note that *Trex1* and *Atrip* polymorphisms do not correlate with the instability phenotype (Figure S9A, B). Further, *Trex1* and *Atrip* striatal mRNA levels are not significantly different in 129 and B6 strains (2-tailed unpaired *t*-test: *p* = 0.73 and *p* = 0.43, respectively) (Figure S9C). While these data do not rule out a role for these genes, these observations make them less compelling candidates as the likely modifiers of strain-specific instability. In contrast, the observation that a “B6-like” haplotype at the *Mlh1* locus is also shared in unstable FVB.*Hdh^Q111^* and DBA.*Hdh^Q111^* strains (Figure S7 and Figure S8) is consistent with the hypothesis that genetic variation at the *Mlh1* locus underlies the difference in striatal *HTT* CAG instability between B6 and 129 strains. This hypothesis also predicts that strains with a “129-like” *Mlh1* haplotype might be more likely to exhibit low *HTT* CAG instability. It is important to note, however, that somatic instability in any particular strain background is likely to be influenced by other genetic variation. Notably, the *Mlh3* gene (chromosome 12), found to be a modifier of CAG instability in this study, does not show genotype differences between B6J and 129S1 strains [44], which are closely related to the B6N and 129S2 strains used here. Therefore, linkage to the *Mlh3* gene would not be expected in our genetic cross. Interestingly, *Msh3* gene variants were recently found to correlate with *HTT* CAG instability in some strains of R6/1 transgenic mice [29]. However, at least for the B6N and 129S2 strains in which we have performed genome-wide QTL mapping, it is clear from the genetic data that any polymorphisms in the *Msh3* gene do not play a significant role in driving these strain-specific differences in somatic expansion of the *Hdh^Q111^* CAG repeat (Figure 2B).

To understand this further we compared non-synonymous *Msh3* SNPs, proposed to underlie the difference in CAG instability between B6 (high instability) and BALB/cJ (low instability) R6/1 mice [29], in strains (B6, 129, FVB and DBA) for which we had quantitative measures of *Hdh^Q111^* striatal instability (Figure S8). Notably B6-BALB/cJ SNPs that are present in 129 and that might be predicted to contribute to low instability in *Hdh^Q111^* mice (those in exons 2, 3 and 7) are also present in unstable FVB and DBA strains (Figure S10A). This suggests that these SNPs are unlikely to contribute to the differences in *Hdh^Q111^* CAG instability between B6 and 129 striata. We also note a very high degree of B6 versus BALB/cJ genetic variation relative to B6 versus 129 genetic variation at the *Msh3* locus (Figure S10B), suggesting the possibility that the apparently complete CAG repeat stabilization in BALB/cJ.R6/1 mice [29] is driven by a *Msh3* polymorphism(s) present in BALB/cJ but not in 129. It is also noteworthy that a single 129 allele increases the instability of the R6/1 CAG repeat in BALB/129 heterozygotes, consistent with higher levels of MSH3 in 129 mice than in BALB/cJ mice [29]. Despite possible locus-specific (*Hdh^Q111^* versus R6/1 mice) and sub-strain differences, the data presented here and previously [29] suggest that the combination of genetic variants in *Mlh1*, *Msh3*, and potentially other MMR genes that are present in any particular mouse strain may determine the rate of CAG expansion in certain tissues.

Given that MLH1 protein levels correlate with striatal expansion in B6 and 129 strains and that the activity of MLH1-dependent DNA repair in cell-free assays is dose-dependent, it is more than plausible to hypothesize that the reduced levels of *Mlh1* expression in 129 mice play an important role in determining the reduced somatic CAG instability observed in *Hdh^Q111^* mice in this genetic background. Given the finding that *Mlh1* is an enhancer of nuclear huntingtin immunostaining, it is also possible that the lower levels of MLH1 in 129 mice contribute to the slowed nuclear huntingtin and inclusion phenotypes previously identified in 129.*Hdh^Q111^* ^/+^ mice compared to B6.*Hdh^Q111/+^* mice [17]. Further unbiased genetic studies would be needed to identify the modifier gene(s) that contribute to these phenotypes. It is worth noting that a number of other studies support a role for the levels or stoichiometries of DNA repair proteins in trinucleotide repeat instability [43], [54]–[57].

Expression analyses of MLH1 mRNA and protein in B6 and 129 strains (Figure 9 and Figure 10) indicate that strain-specific polymorphisms may act at both transcriptional and post-transcriptional levels. Assuming that B6.*Mlh1* ^+/−^ and B6.*Mlh1* ^+/+^ striata display comparable levels of instability at 10 weeks of age, as seems likely from the similar levels of instability in B6.*Mlh1* ^+/−^ and B6.*Mlh1* ^+/+^ mice at 22 weeks of age (Figure 4), a comparison of somatic instability and MLH1 protein in B6.*Mlh1* ^+/+^, 129.*Mlh1* ^+/+^ and B6.*Mlh1* ^+/−^ striata (Figure 1, Figure 4, Figure S1, and Figure 9) suggests that there may be a threshold level of MLH1 protein below which MLH1-dependent process(es) that mediate expansion are compromised. In this scenario, MLH1 protein in B6.*Mlh1* ^+/−^ mice, although reduced compared to that in B6.*Mlh1* ^+/+^ mice, exceeds this threshold, with the result that the *HTT* CAG repeat remains unstable. In 129 mice, the MLH1 protein level falls below the threshold and the *HTT* CAG repeat is consequently stabilized. Alternatively, it is possible that reduced MLH1 protein alone is insufficient to explain the *HTT* CAG repeat stabilization in 129 mice, but that a functional alteration of the 129 protein acts in concert with the reduced expression level to decrease *HTT* CAG expansion efficiency. Although we were unable to demonstrate any difference in activity between B6 and 129 recombinant MLH1 proteins in cell-free MMR assays (Figure 8 and Figure S12), these assays may not be sufficiently sensitive to detect subtle alterations in function. It is also important to note that the MMR ability of MLH1 was only investigated in the context of MutLα-mediated repair. Therefore, taking into account our finding that MLH3 is essential for somatic *HTT* CAG instability *in vivo*, we cannot rule out the hypothesis that B6 and 129 MLH1 proteins may have dissimilar MutLγ-mediated repair potential. It is also possible that MLH1 function may differ between B6 and 129 strains in other ways *in vivo* that cannot be captured in the cell-free systems, *e.g.* altered interaction with binding partners. Thus, while our data indicate that MLH1 protein levels are likely to be a driving force in determining the differential *HTT* CAG somatic expansion potential in B6 and 129 strains, phenotypic comparisons between strains at the level of MLH1 mRNA, protein and *HTT* CAG instability, together with the highly polymorphic nature of the *Mlh1* locus, suggest that the genetic architecture underlying the strain-specific differences in instability may be complex.

MLH1 has been found to play a role in CAG repeat instability in a selectable cell-based system [58]. A functional form of MLH1, with an intact ATPase domain, is also required to repair slipped CAG/CTG structures *in vitro* [50] (Figure 8). To our knowledge no role for MLH3 in trinucleotide repeat instability has been previously demonstrated. Here, we show for the first time that both *Mlh1* and *Mlh3* genes enhance *HTT* CAG expansion in a trinucleotide repeat disease mouse model. Our data further consolidate the critical role of MMR genes as enhancers of *HTT* CAG-dependent events [18]–[27], [29] in *Hdh^Q111^* mice. We were unable to determine the effect of loss of *Mlh1* or *Mlh3* on intergenerational instability of the *HTT* CAG repeat in *Hdh^Q111^* mice as *Mlh1* and *Mlh3* null mice are sterile [36], [39]. Interestingly, as with somatic instability, B6.*Hdh^Q111^* mice show a greater degree of intergenerational CAG repeat instability than 129.*Hdh^Q111^* mice [17]. Given evidence suggesting a role for MMR pathways in both somatic and intergenerational repeat instability [18], [23], [59], it is plausible that genetic variation at the *Mlh1* locus also underlies the difference in intergenerational instability between the two strains.

The mechanism(s) by which MMR proteins mediate somatic CAG/CTG expansion is unclear. Importantly, we find that the MutLγ components, MLH1 and MLH3, are as critical to somatic *Hdh^Q111^* CAG expansion as the MutSβ components MSH2 and MSH3 [18], [19], suggesting that MutLγ and MutSβ are involved in the same pathway that promotes CAG/CTG expansion. While a role for proteins downstream of MutL complexes in somatic CAG/CTG expansion has not been demonstrated to date, the requirements for MLH1 and MLH3 indicate that the generation of somatic *Hdh^Q111^* CAG expansions requires active engagement of the MMR machinery, in contrast to a model whereby expansions occur due to the inability of MutSβ-CAG/CTG repeat binding to execute coupling to downstream effector functions [25], [60]. Our findings also argue against MutSβ-mediated expansion arising via other pathways that are MutL-independent, such as single strand annealing [23], [61]. Our results support previously published studies in mouse models of DM1 in which somatic expansion of the CTG repeat was reduced in *Pms2* null mice [24] or inhibited in mice deficient in MSH2's ATPase function, which is required for MutL complexes recruitment [27]. Recruitment of MutL complexes is a required step for subsequent enzymatic processing of the DNA mismatch [37], [38]. An essential function of MutLα is the activation of the latent endonuclease activity of PMS2 [62], which, interestingly, is activated by extrahelical CAG/CTG repeats *in vitro* [63]. It would therefore be of interest to determine whether MLH3's putative endonuclease domain [62] is required for CAG expansion *in vivo*.

The MMR pathway, as traditionally described, is employed to repair errors that are incurred during DNA replication. However, there is increasing evidence that MMR proteins play various roles in the absence of DNA replication and participate in a variety of other pathways, distinct from MMR [64]–[69]. Recently, a promutagenic noncanonical MMR pathway has been described, which occurs in multiple cell types, is independent of DNA replication and is activated by DNA lesions rather than mismatches [70]. The findings that MMR proteins are required for, rather than protect against somatic CAG/CTG instability, that repeat expansions occur in postmitotic cells [10], [33], [71] and that expansions in neurons require MSH2 [20], suggest that CAG/CTG repeat expansion may arise via a noncanonical MMR pathway(s).

**Figure 11:**
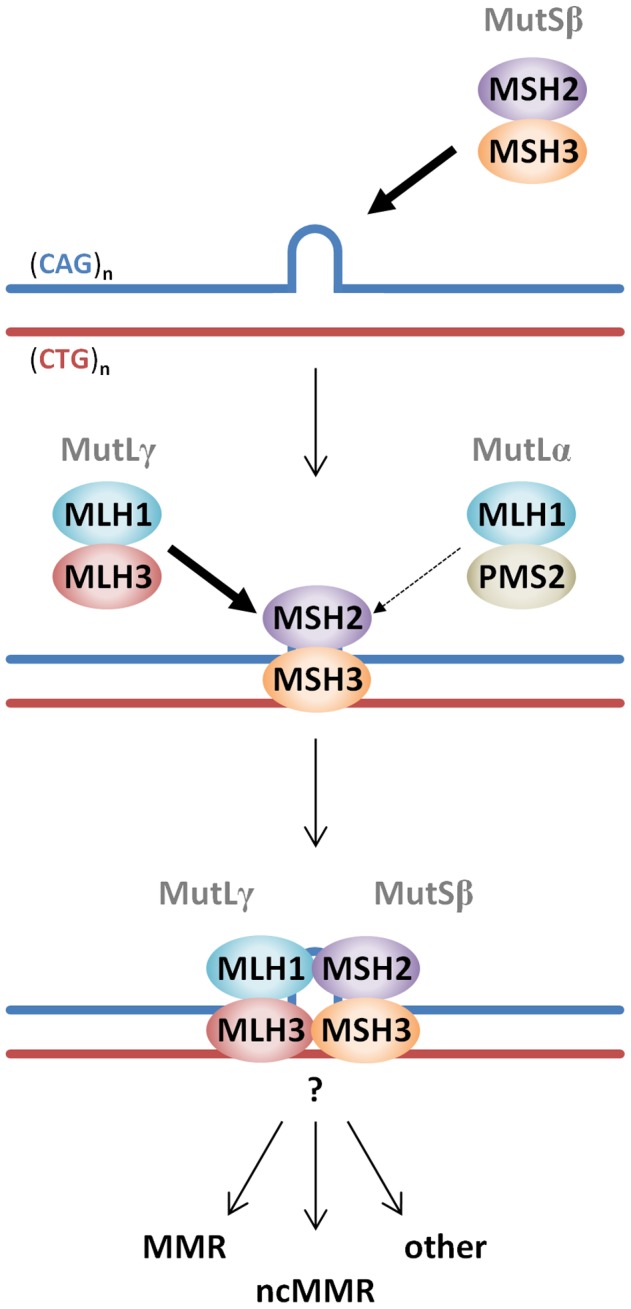
Proposed model of MutS and MutL-dependent events leading to CAG•CTG somatic instability. CAG•CTG repeat structures are initially recognized by the MutSβ (MSH2-MSH3) complex [25], [98]. The loop in the CAG•CTG repeat tract represents a short slip-out, previously identified as the main substrate for MMR protein-dependent repair of CAG•CTG structures in cell free systems [50], [51]. However, the nature of the putative CAG•CTG structure(s) that leads to MutS and MutL-dependent somatic instability *in vivo* is unknown. Following ATP hydrolysis by DNA-bound MutSβ [27], a MutLγ (MLH1–MLH3) heterodimer is preferentially recruited to the complex (thick arrow) over the MutLα (MLH1-PMS2) heterodimer (thin arrow). The total absence of *HTT* CAG expansion in *Mlh3* ^−/−^ mice suggests that PMS2 plays no role at all in this process. However, PMS2 has been shown to play a role in the expansion of CTG repeats in a DM1 mouse model [24], suggesting that these events may be genetic locus and/or mouse strain dependent. Following MutLγ binding, various pathways, *e.g.* canonical mismatch repair (MMR), noncanonical mismatch repair (ncMMR) and/or other DNA repair processes may be engaged and process the repeats such that they ultimately undergo expansion. Other members of alternative DNA repair pathways, namely OGG1, XPA and NEIL1 have been directly implicated in CAG/CTG somatic instability in mice [76]–[78], however, how these proteins intersect with MMR protein-dependent pathways has yet to be demonstrated.

With regard to potential mechanisms of CAG expansion it is of interest that MSH3 and MLH3 appear to play relatively minor roles in classical MMR inasmuch as *Msh3* and *Mlh3* deficiencies result in weak mutator phenotypes and relatively low cancer predisposition phenotypes [42], [72]–[75]. In strong contrast, loss of either of these two proteins has a major impact on CAG/CTG expansion. Conversely, MSH6 and PMS2 play prominent roles in classical MMR [72]–[74]. However, MSH6 is either unnecessary for, or plays a very minimal role in mediating somatic CAG/CTG expansions [19], [22], [25], and knockout of *Pms2* had a moderate effect of CTG expansion in DM1 mice [24], implicating a role for different MLH1 partners. In the present study the complete absence of *HTT* CAG expansion in *Hdh^Q111^* ^/+^ *Mlh3* null mice argues against a role for PMS2 in generating expansions in these mice. Further genetic crosses in both DM1 and *Hdh^Q111^* mice would be needed to determine whether the relative contributions of *Pms2* and *Mlh3* genes in the two mouse models depends on the genomic locus of the repeat and/or strain background. While we do not expect PMS2 levels to be altered in *Mlh3* knockout mice [74], additional experiments are needed in *Mlh3* and *Pms2* knockout mouse tissues to determine whether any compensatory changes in PMS2 or MLH3 proteins, respectively, occur. However, overall, the data thus far indicate that MLH3 is a more significant player than PMS2 in CAG/CTG expansion and suggest that CAG/CTG repeats may preferentially engage a pathway(s) involving MutSβ and MutLγ complexes, as illustrated in Figure 11.

Given the overlapping roles of MMR proteins in both DM1 and HD mouse models [18]–[27], [29], the findings in the present study are predicted to be directly relevant both to DM1 and likely other CAG/CTG repeat expansion diseases. However, subtle qualitative and quantitative differences in the effects of MMR genes in the various mouse models suggest a potential modulatory role for the *cis*-sequence surrounding the repeat. In addition, proteins in base excision repair and nucleotide excision repair pathways have also been found to play role in mouse models of CAG/CTG expansion disorders [76]–[78]. Further studies will be needed to determine how the various DNA repair proteins might intersect to mediate CAG/CTG expansion and the extent to which their effects might depend on genomic context.

In summary, we have taken both unbiased and candidate gene approaches towards understanding the factors that underlie the instability of the *HTT* CAG repeat. Unbiased linkage mapping in congenic *Hdh^Q111^* mice indicated *Mlh1* as a potential genetic modifier of strain-specific *HTT* CAG instability. Subsequent candidate gene approaches demonstrated both *Mlh1* and *Mlh3* as critical novel modifiers of *HTT* CAG instability. The identification of *Mlh1* and *Mlh3* as modifiers of CAG instability in *Hdh^Q111^* mice suggests that variation in the human *MLH1* and *MLH3* genes may contribute to differences in somatic *HTT* CAG expansion that occurs between HD patients [9], [11]. Further, given their minor roles in human tumorigenesis, both MLH3 and MSH3 currently stand as the most promising targets of the MMR proteins that have been identified as modifiers of the *HTT* CAG pathogenic process to date. Further delineation of the factors involved in somatic instability and the pathway(s) involved are likely to increase the ability to specifically intervene in the process of CAG/CTG expansion in HD as well as other trinucleotide repeat disorders.

## Materials and Methods

### Mice

Ethics statement: All animal procedures were carried out to minimize pain and discomfort, under approved IACUC protocols of the Massachusetts General Hospital and Cornell University. Congenic *Hdh^Q111^* strains on C57BL/6NCrl (B6N), 129S2/SvPasCrlf (129) and FVB/NCrl (FVB) genetic backgrounds have been previously described [17]. In addition we generated *Hdh^Q111^* strains on DBA/2J (DBA) and C57BL/6J (B6J) backgrounds by repeated backcrossing of CD1.*Hdh^Q111^* ^/+^ mice [15] for at least 10 generations. To map genetic modifiers of somatic *HTT* CAG instability we generated (B6Nx129).*Hdh^Q111/+^* and (B6Nx129).*Hdh^+/+^* F1 mice which were subsequently intercrossed to generate (B6Nx129).*Hdh^Q111/+^* F2 progeny. B6.*Mlh1* knockout mice (B6N) [36] were crossed with B6N.*Hdh^Q111^* mice, and B6.*Mlh3* knockout mice (B6J) [39] were crossed with B6J.*Hdh^Q111^* mice to generate B6.*Hdh^Q111/+^* mice heterozygous for the respective DNA repair mutation. These mice were then intercrossed to generate B6.*Hdh^Q111/+^* littermates that were wild-type (+/+), heterozygous (+/−) or homozygous mutant (−/−) for the respective DNA repair gene. For reasons of simplicity, both B6N and B6J will be referred to as B6 unless otherwise specified. *Mlh1* knockout mice were also generated on the 129 background by repeated backcrossing of B6.*Mlh1^+/−^* mice for 4 generations. These mice were then intercrossed to generate 129.*Mlh1^+/+^*, 129.*Mlh1^+/−^* and 129.*Mlh1^−/−^* littermates. Animal husbandry was performed under controlled temperature and light/dark cycles.

### Genotyping and *HTT* CAG repeat analysis

Genomic DNA was isolated from tail biopsies at weaning for routine genotyping analysis or from adult tissues (fresh frozen or fixed as below) for somatic instability analysis, using the PureGene DNA isolation kit (Qiagen). Routine genotyping was carried out as previously described [19], [36], [39]. The size of the *HTT* CAG repeat was determined using a human-specific PCR assay that amplifies the *HTT* CAG repeat from the knock-in allele but does not amplify the mouse sequence [79]. The forward primer was fluorescently labeled with 6-FAM (Applied Biosystems) and products were resolved using the ABI 3730xl DNA analyzer (Applied Biosystems) with GeneScan 500 LIZ as internal size standard (Applied Biosystems). GeneMapper v3.7 (Applied Biosystems) was used to generate CAG repeat size distribution traces. Repeat size was determined from the peak with the greatest intensity in the GeneMapper trace from the tail biopsy (“main allele”). CAG repeat instability index was calculated as previously described [32]. Briefly, the highest peak in each trace was used to determine a relative threshold of 20% and peaks falling below this threshold were excluded from analysis. Peak heights normalized to the sum of all peak heights were multiplied by the change in CAG length of each peak relative to the main allele size in tail. These values were summed to generate an instability index, which represents the mean CAG repeat length change in the population of cells being analyzed. Statistical comparisons of instability indices were carried out using 2-tailed unpaired *t*-tests.

### Quantitative trait loci (QTL) mapping

Somatic CAG instability indices were determined in the striatum of 69 10-week-old (B6x129).*Hdh^Q111/+^* F2 mice, as described above. These F2 intercross mice were originally genotyped using a panel of 117 SNPs that distinguishes between C57BL/6J and 129S1/SvImJ strains (Figure S3 and Table S1) [80]. An additional set of 30 SNPs was subsequently used to add resolution to the analysis (Figure S3 and Table S1), particularly at the chromosome 9 QTL, including two markers inside the *Mlh1* gene (dbSNP rs30131926 and rs30174694); as well as to specifically investigate the *Msh2* (dbSNP rs33609112 and rs49012398) and *Msh3* (dbSNP rs29551174) genes. Linkage analysis was performed using Mapmaker/QTL [81]–[84], with striatal *HTT* CAG instability indices as quantitative traits. A threshold LOD-score of 4.3 was considered for the identification of significant QTLs [85]. A QTL 95% confidence interval was determined by using the 2-LOD-dropoff method [35], [86].

### Identification and analyses of polymorphisms

Polymorphisms at the *Mlh1* locus were investigated between C57BL/6NCrl (B6N), 129S2/SvPasCrlf (129S2), FVB/NCrl (FVB) and DBA/2J (DBA) genetic strains by standard DNA Sanger sequencing. PCR products were generated using *Taq* DNA polymerase (Qiagen) with DNA extracted from tail as template. A combination of primer pairs (Table S2) was used to screen the complete coding sequence of *Mlh1* as well as its immediate 5′ and 3′ flanking regions (2.6 kb and 2 kb respectively) by sequencing both sense and antisense strands. Polymorphisms were validated in two animals from each genetic strain. We also utilized an online database for the Mouse Genomes Project (http://www.sanger.ac.uk/resources/mouse/genomes), provided by the Wellcome Trust Sanger Institute. This database was derived from whole genome sequencing of 17 different genetic mouse strains [44], [45], including C57BL/6NJ (B6NJ), 129S1/SvImJ (129S1), DBA/2J (DBA) and FVB/NJ (FVB). We used this database to retrieve information on SNPs, short indels and structural variants over a 64 kb region encompassing the *Mlh1* gene (chr9:111,223,496–111,287,496), as well as at the *Mlh3* (chr12:85,234,529–85,270,591), *Msh3* (chr13:92,201,881–92,365,003), and *Trex1*/*Atrip* loci (chr9:109,057,933–109,074,124; GRCm38/mm10 assembly). The average genome-wide variation between B6 and 129 was determined using the total number of SNPs and indels reported in this database (B6J versus 129S1) relative to the GRCm38/mm10 genome size (chromosomes 1–19 and X). The relative density of polymorphisms between B6 and 129 was determined by binning genome-wide SNPs and indels into 64 kb regions (the same size as the *Mlh1* genomic region analyzed) and the mean density of polymorphisms/kb determined over each of the 64 kb bins. For reasons of simplicity, both B6N and B6NJ are referred to as B6, 129S1 and 129S2 are referred to as 129, and FVB/NCrl and FVB/NJ are referred to as FVB, unless otherwise specified.

### Immunohistochemistry

Immunostaining was carried out with polyclonal anti-huntingtin antibody EM48 [87] on 7 µm paraffin-embedded coronal sections of periodate-lysine-paraformaldehyde (PLP)-perfused mouse brains, as previously described [17]. Diffuse EM48 immunostaining was quantified as a “staining index” that captures both the nuclear staining intensity and the number of immunostained nuclei, as described previously [17]. Statistical comparisons of staining indices were carried out using 2-tailed unpaired *t*-tests.

### Cell-free mismatch repair assays

Total RNA was isolated from the striatum of wild-type B6 and 129 mice using Trizol (Life Technologies) by mechanical grinding with disposable pestle and cDNA was then prepared using the SuperScript III First-Strand Synthesis SuperMix for qRT-PCR (Invitrogen). Full-length *Mlh1* cDNAs were amplified by PCR (for primers used see Table S2) using Phusion High-Fidelity DNA polymerase (New England Biolabs), and were subsequently cloned between the unique NcoI and XhoI sites of a modified pFastBac1 baculovirus expression vector [88], so that the resulting recombinant MLH1 proteins would carry N-terminal FLAG and 6xHis epitope tags. *Mlh1* cDNA pFastBac1 constructs were fully verified by DNA sequence analysis confirming the presence of all B6-129 SNPs (for primers used see Table S2). The wild-type human *MLH1* cDNA (hMLH1-WT) baculovirus expression vector [49] was used to generate a mutant h*MLH1* cDNA construct carrying the 129-like Ile residue at aa192 (hMLH1-F192I) by site directed mutagenesis. Mouse and human recombinant MLH1 proteins were independently co-expressed with human PMS2 and purified using a baculovirus expression system to near homogeneity (Figure S11), as previously described [49]. Protein concentrations were determined spectrophotometrically and confirmed by polyacrylamide gel electrophoresis (PAGE). Repair of a single base mismatch by MLH1 was investigated as previously described [49]. In essence, repair of single base mismatch (G-T) substrate containing a 5′ nick was assessed using HeLa or MutLα-deficient HCT116 [89] nuclear protein extracts (100 ng) complemented with equal amounts of purified MutLα protein complexes: hMLH1.WT-hPMS2, hMLH1.F192I-hPMS2, mMLH1.B6-hPMS2 or mMLH1.129-hPMS2 (100 ng). Note that as mMLH1-hPMS2 was functional in this well-established human-based assay, consistent with previous mixed yeast-human MMR assays [90]–[92], we compared B6 and 129 MLH1 proteins in a mixed mouse-human MutLα complex, avoiding the need to introduce mouse PMS2 as another assay variable. Single base mismatch repair was analyzed by agarose gel electrophoresis followed by ethidium bromide staining [49]. Repair of a single trinucleotide repeat slip-out by MLH1 was investigated as previously described [50]. In summary, repair of single CTG slip-out substrates (CAG)_47_•(CTG)_48_ containing a 5′ nick was assessed using HeLa or MutLα-deficient HEK293T [42], [93] whole cell extracts (120–180 ng) complemented with equal amounts of purified hMLH1.WT-hPMS2, mMLH1.B6-hPMS2 or mMLH1.129-hPMS2 complexes (100 ng), or with increasing amounts of mMLH1.B6-hPMS2 or mMLH1.129-hPMS2 complexes (5, 25 and 100 ng). This experiment with increasing concentrations was reproduced three times. Repair of CTG slip-outs was analyzed by Southern blotting. For both MMR assays, intensity of fragments was determined by densitometry and repair activity was determined as the intensity of repair fragments in proportion to the total intensity of all fragments [49], [50]. Statistical comparison between mMLH1.B6-hPMS2 and mMLH1.129-hPMS2 repair efficiency was carried out using 2-tailed unpaired *t*-tests. MutLα dose-dependency of CTG slip-outs repair was determined by linear regression. The HEK293T cell line was a gift from Dr. G. Plotz. HeLa cells were from the National Cell Culture Center, National Center for Research Resources, National Institutes of Health.

### mRNA and protein expression analyses

mRNA and protein expression was investigated in frozen striatum samples from 10-week-old mice (B6.*Mlh1^+/+^*, *n* = 3; 129.*Mlh1^+/+^*, *n* = 3; B6.*Mlh1^+/−^*, *n* = 3; and B6.*Mlh1^−/−^*, *n* = 1), with the striatum from one hemisphere being used for mRNA analysis by qRT-PCR and the other being used for protein analysis by western blotting. Total RNA extraction and first-strand cDNA synthesis were performed as described above. Relative qRT-PCR was performed on a LightCycler 480 Real-Time PCR System (Roche) using TaqMan Gene Expression Master Mix (Applied Biosystems) and TaqMan Gene Expression Assays (Applied Biosystems) for: *Mlh1* (exons 4–5, Mm01248478_m1; exons 11–12, Mm00503449_m1; exons 18–19, Mm00503455_m1), *Trex1* (Mm00810120_s1), and *Atrip* (Mm00555350_m1). Relative mRNA expression levels were determined using the 2^−ΔΔCp^ method [94] by normalization to the housekeeping gene *Actb* (Mm00607939_s1). Each sample was run in triplicates and a total of 2 runs were performed. Protein lysates were prepared in RIPA buffer supplemented with 5 mM EDTA and protease inhibitors (Halt Protease Inhibitor Cocktail, Thermo Scientific) by mechanical grinding with disposable pestle and two 10-second sonication pulses (Branson sonifier, power level 3.5), on ice. The homogenates were kept on ice for 30 min and then clarified by centrifugation at 4°C for 30 minutes at 14000 rpm. Protein concentration was determined using the DC protein assay kit (Bio-Rad). Western blot analysis was carried out by resolving protein extracts (50 µg) on 4–12% Bis-Tris polyacrylamide gels (NuPAGE, Life Technologies). All samples were run in the same gel and a total of 2 gels were run. Rabbit polyclonal antibody against the C-terminal end of MLH1 (1∶200; sc-582, Santa Cruz Biotechnology) and mouse monoclonal antibody against α-tubulin (1∶1,000; #3873, Cell Signaling Technologies) were used as primary antibodies and horseradish peroxidase-conjugated goat anti-rabbit and anti-mouse (1∶10,000; NA934VS and NA931VS respectively, Amersham) were used as secondary antibodies. Signals were visualized using enhanced chemiluminescence (ECL) detection system (Thermo Scientific). Densitometric analysis of protein levels was performed using UN-SCAN-IT software (Silk Scientific Corp.). Following background subtraction, MLH1 protein levels were normalized to α-tubulin, and determined relative to B6.*Mlh1^+/+^* levels. Statistical comparisons of mRNA and protein levels were carried out using 2-tailed unpaired *t*-tests.

### *Mlh1*–luciferase reporter assays

The immediate 5′- and 3′-flanking regions of *Mlh1* were amplified by PCR from both B6 and 129 genomic DNA (for primers used see Table S2) using Phusion High-Fidelity DNA polymerase (New England Biolabs). The immediate 5′-flanking region of *Mlh1* (2,441 bp) was cloned upstream of the firefly luciferase reporter in pGL4.20 (Promega) between the unique KpnI and NheI sites. Progressively smaller segments of the immediate 3′-flanking region of *Mlh1* (1,676 bp, 1,280 bp, 591 bp and 205 bp) were cloned downstream of the firefly luciferase reporter in pGL3-Promoter (Promega) between the unique XbaI and BamHI sites. Additional “swap” constructs were also generated for the immediate 3′-flanking region of *Mlh1* (1,676 bp) by dividing this region into 3 distinct subdomains (5′-3′: 530 bp, 438 bp and 708 bp; using PacI and KpnI) and replacing individual subdomains from the B6 3′-flanking region of *Mlh1* with the corresponding 129 subdomain. “Swap” constructs were cloned downstream of the firefly luciferase reporter in pGL3-Promoter (Promega) at the unique XbaI site. *Mlh1*–luciferase reporter constructs were fully verified by DNA sequence analysis, confirming the presence of all B6-129 SNPs (for primers used see Table S2). Individual *Mlh1*–firefly luciferase reporter constructs were co-transfected (Lipofectamine LTX, Invitrogen) with the *Renilla* luciferase reporter control pGL4.74 (Promega) into wild-type mouse immortalized striatal cells [95]. The transfected cells were cultured for 36–48 hours and luciferase expression was subsequently quantified using the Dual-Luciferase Reporter Assay System (Promega) on a microplate luminometer (MicroLumat Plus LB96V, Berthold Technologies). Analogous B6 and 129 *Mlh1*–luciferase constructs were investigated in the same experiment in triplicate. The relative luciferase activity was calculated by normalizing firefly luminescence to the internal *Renilla* signal and determined relative to the corresponding B6 construct. Statistical comparison of relative luciferase activity between analogous B6 and 129 *Mlh1*–luciferase constructs was carried out using 2-tailed unpaired *t*-tests.

## Supporting Information

Figure S1 Somatic *HTT* CAG instability in 22-week-old B6.*Hdh^Q111^* ^/+^ and 129.*Hdh^Q111^* ^/+^ mice. Representative GeneMapper profiles of *HTT* CAG repeat size distributions in the tail and striatum of 22-week-old B6.*Hdh^Q111/+^* and 129.*Hdh^Q111/+^* mice, highlighting the high degree of somatic instability in B6 mice versus the reduced contribution of the 129 genetic background to somatic *HTT* CAG repeat expansions, as previously described [17]. Tail and striatum: B6.*Hdh^Q111/+^*, CAG112; 129.*Hdh^Q111/+^*, CAG110.
(TIF)

Figure S2 CAG repeat lengths of 10-week-old *Hdh^Q111/+^* mice on different genetic backgrounds. Graphical representation of CAG repeat lengths of individual mice used in this study, grouped according to genetic background and color-coded based on genotype. F2 mice are color-coded by *Mlh1* genotype. Blue: homozygous for B6 alleles; red: homozygous 129; green: heterozygous B6/129; purple: failed genotype. Constitutive *Hdh* CAG repeat lengths were determined from tail samples. dbSNP markers located within *Mlh1* gene: rs30131926 and rs30174694 (concordant genotypes detected with both markers). B6.*Hdh^Q111/+^*, *n* = 10; 129.*Hdh^Q111/+^*, *n* = 12; (B6x129).*Hdh^Q111/+^* F1, *n* = 11; (B6x129).*Hdh^Q111/+^* F2, *n* = 69. Horizontal bars represent the mean CAG repeat length of respective group.
(TIF)

Figure S3 Chromosomal distribution of genetic markers used for QTL analysis. An initial panel of 117 SNPs (green triangles) that distinguish between B6 and 129 strains was used to perform linkage analysis, resulting in the identification of a QTL in chromosome 9 (Figure S4). An additional set of 30 SNPs (red triangles) was subsequently used to enhance resolution at this QTL and improve overall genome coverage, but also to specifically investigate the *Mlh1*, *Msh2* and *Msh3* genetic loci (Figure 3). Marker chromosomal positions and dbSNP references can be found in Table S1.
(TIF)

Figure S4 Preliminary mapping of QTL associated with striatal CAG instability. Preliminary linkage analysis in 10-week-old (B6x129).*Hdh^Q111/+^* F2 mice (*n* = 69) identified a single QTL on chromosome 9 with a LOD score of ∼11. The red dashed line represents the threshold (LOD = 4.3) considered for the identification of significant QTLs [85]. The coordinates (cM) of the 117 genetic markers used are represented by open triangles.
(TIF)

Figure S5 Preliminary mapping of QTL on chromosome 9 implicates numerous genes, including the MMR gene *Mlh1*. Genome-wide linkage analysis using an initial set of 117 SNPs mapped a single QTL on chromosome 9 strongly linked to striatal CAG instability (Figure S4). A 95% confidence interval was determined by using the 2-LOD-dropoff method [35], [86], implicating a genomic region of approximately 39 Mb (chr9:84,495,988–123,231,477; GRCm38/mm10) that contained numerous genes (∼420), including the MMR gene *Mlh1*.
(TIF)

Figure S6 Fine-mapping of chromosome 9 QTL significantly narrowed down the implicated genomic region and number of candidate genes. Follow-up genome-wide linkage analysis with additional genetic markers mapped a single QTL on chromosome 9 strongly linked to striatal CAG instability (Figure 3). A 95% confidence interval was determined by using the 2-LOD-dropoff method [35], [86], narrowing down the implicated region to approximately 5 Mb (chr9:107,982,655–113,057,967; GRCm38/mm10). In addition to *Mlh1*, the implicated genomic region contains numerous genes (∼100).
(TIF)

Figure S7 Genetic variation at the *Mlh1* locus between different mouse strains. (A) Nonsynonymous polymorphisms identified at the *Mlh1* locus in the unstable C57BL/6NCrl, FVB/NCrl and DBA/2J *Hdh^Q111^* strains, versus the more stable 129S2/SvPasCrlf *Hdh^Q111^* strain. (B) Distribution of polymorphisms identified between C57BL/6NJ, 129S1/SvImJ, FVB/NJ and DBA/2J mouse strains at a 64 kb genomic region encompassing the *Mlh1* gene (chr9:111,223,496–111,287,496; GRCm38/mm10) using information from the Mouse Genomes Project [44], [45]. Red, nonsynonymous SNPs (nsSNPs); blue, SNPs; green, short indels; purple, structural variants (SV).
(TIF)

Figure S8 Higher levels of somatic *HTT* CAG instability in B6, FVB and DBA mice compared to 129. (A) Representative GeneMapper profiles of *HTT* CAG repeat size distributions in the tail and striatum of 10-week-old C57BL/6NCrl (B6), 129S2/SvPasCrlf (129), FVB/NCrl (FVB) and DBA/2J (DBA) *Hdh^Q111/+^* mice, emphasizing the contribution of genetic background to somatic *HTT* CAG repeat expansion, as previously described [17]. Tail: B6.*Hdh^Q111/+^*, CAG117; 129.*Hdh^Q111/+^*, CAG108; FVB.*Hdh^Q111/+^*, CAG122; DBA.*Hdh^Q111/+^*, CAG115 (B) Quantification of CAG instability index reveals significantly higher levels of somatic *HTT* CAG instability in the striatum of B6, FVB and DBA *Hdh^Q111^* ^/+^ mice compared to 129.*Hdh^Q111^* ^/+^ mice. B6.*Hdh^Q111/+^*, *n* = 10, CAG116.9±1.2SD; 129.*Hdh^Q111/+^*, *n* = 12, CAG110.9±1.2SD; FVB.*Hdh^Q111/+^*, *n* = 3, CAG123.7±2.1SD; DBA.*Hdh^Q111/+^*, *n* = 3, CAG115.7±1.2SD; Bar graphs represent mean ±SD; ***, *p*<0.001; ****, *p*<0.0001.
(TIF)

Figure S9 Comparison of *Trex1* and *Atrip* genes in different mouse strains. (A) Nonsynonymous polymorphisms identified at the *Trex1* and *Atrip* locus in the unstable B6, FVB and DBA *Hdh^Q111^* strains, versus the more stable 129 *Hdh^Q111^* strain. (B) Distribution of polymorphisms identified between B6, 129, FVB and DBA mouse strains at a 16 kb genomic region containing the *Trex1* and *Atrip* genes (chr9:109,057,932–109,074,124; GRCm38/mm10) using information from the Mouse Genomes Project [44], [45]. Red, nonsynonymous SNPs (nsSNPs); blue, SNPs; green, short indels. (C) Quantification of *Trex1* and *Atrip* mRNA levels in the striatum of B6 and 129 10-week-old mice (*n* = 3) by TaqMan-based qRT-PCR. mRNA levels were determined relative to the housekeeping gene *Actb*. Bar graphs represent mean ±SD.
(TIF)

Figure S10 Genetic variation at the *Msh3* locus between different mouse strains. (A) Nonsynonymous polymorphisms identified at the *Msh3* locus in B6, BALB, 129, FVB and DBA mouse strains. (B) Distribution of genetic polymorphisms identified between C57BL/6NJ, BALB/cJ, 129S1/SvImJ, FVB/NJ and DBA/2J mouse strains across a region encompassing the *Msh3* gene (chr13:92,201,881–92,365,003; GRCm38/mm10) using information from the Mouse Genomes Project [44], [45]. Red, nonsynonymous SNPs (nsSNP); blue, SNPs; green, short indels; purple, structural variants (SV).
(TIF)

Figure S11 Purified MutLα protein complexes used for cell-free MMR assays. Human and mouse MLH1 proteins from B6 and 129 strains were independently co-expressed with human PMS2 protein in a baculovirus expression system. Purified MutLα complexes were analyzed by polyacrylamide gel electrophoresis and coomassie blue staining.
(TIF)

Figure S12 B6 and 129 MLH1 proteins show similar ability to repair single base mismatches in a cell-free MMR assay. Repair of a single base mismatch (G-T) containing 5′ nick using HeLa or HCT116 (MutLα-deficient) nuclear extracts complemented with equal amounts of purified MutLα protein complexes: hMLH1.WT-hPMS2, hMLH1.F192I-hPMS2, mMLH1.B6-hPMS2 or mMLH1.129-hPMS2. Both B6 and 129 MLH1 proteins show ability to repair the mismatch when in a complex with hPMS2, with no overt difference in repair efficiency being observed between the two (lanes 5 and 6). Likewise, introduction of the 129-like F192I mutation in the human MLH1 protein had no discernible effect in mismatch repair efficiency (lane 4).
(TIF)

Figure S13 Additional analyses of *Mlh1* mRNA levels in B6 versus 129 strains. (A) Quantification of *Mlh1* mRNA levels in the striatum of B6 and 129 10-week-old mice (*n* = 3) using TaqMan assays probing 3 distinct regions of the primary *Mlh1* transcript (exons 4–5, 11–12, and 18–19) confirmed consistently reduced *Mlh1* mRNA levels in the striata of 129 mice, to approximately 50% of B6 levels. (B) The levels of *Mlh1* mRNA (exons 11–12) were also reduced in other tissues of 129 mice (cerebellum, liver, jejunum and ileum), to between 25% and 50% of B6 levels (*n* = 2). *Mlh1* mRNA levels were determined by TaqMan-based qRT-PCR relative to the housekeeping gene *Actb*. Bar graphs represent mean ±SD. **, *p*<0.01.
(TIF)

Figure S14 Additional analyses of MLH1 protein by western blot. (A) Representative western blot used for quantification of MLH1 protein levels in the striatum of B6.*Mlh1^+/+^*, 129.*Mlh1^+/+^* and B6.*Mlh1^+/−^* 10-week-old mice as represented in Figure 9C. The horizontal dashed line represents where the blot was cut (∼60 kDa). The top panel of the blot was probed against MLH1, while the bottom was probed against α-tubulin as loading control. (B) MLH1 western blot of cortex samples from 10-week-old B6 and 129 mice on different *Mlh1* genetic backgrounds.
(TIF)

Table S1 List of genetic markers used for QTL mapping.
(PDF)

Table S2 List of primers used.
(PDF)
