# Supplementary figures and images for: Mismatch Repair Genes Mlh1 and Mlh3 Modify CAG Instability in Huntington's Disease Mice: Genome-Wide and Candidate Approaches
Source: PLoS Genet. 2013 Oct 31;9(10):e1003930. doi: 10.1371/journal.pgen.1003930 (PMC3814320; doi:10.1371/journal.pgen.1003930)

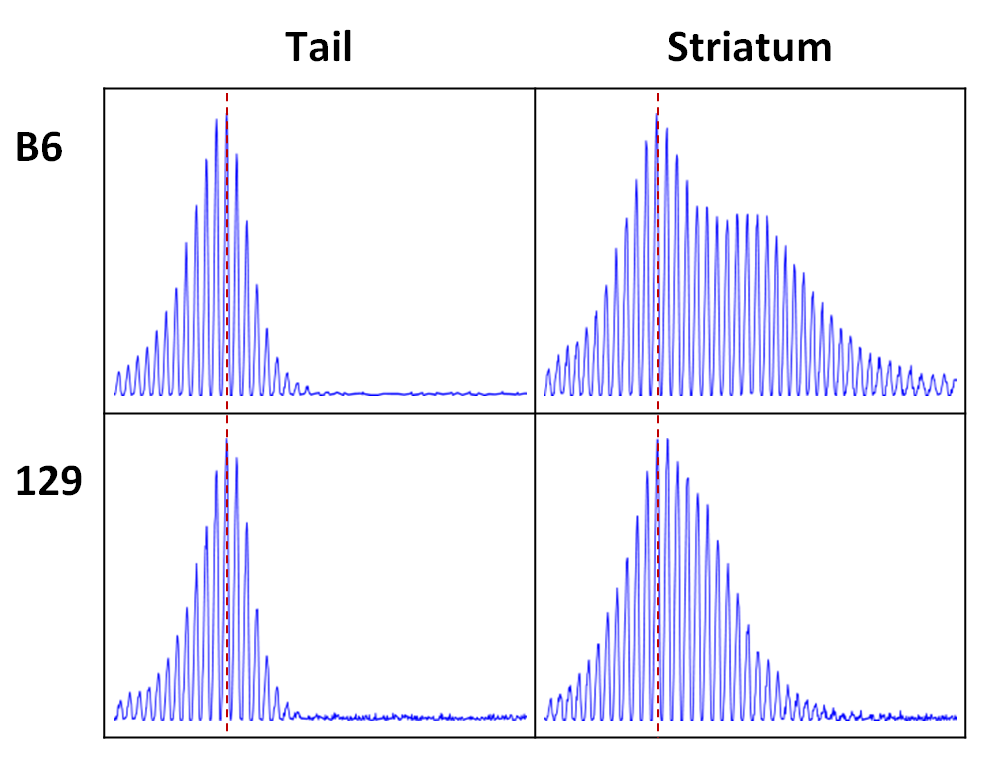

Supplement: Figure S1 — Somatic HTT CAG instability in 22-week-old B6.HdhQ111 /+ and 129.HdhQ111 /+ mice. Representative GeneMapper profiles of HTT CAG repeat size distributions in the tail and striatum of 22-week-old B6.HdhQ111/+ and 129.HdhQ111/+ mice, highlighting the high degree of somatic instability in B6 mice versus the reduced contribution of the 129 genetic background to somatic HTT CAG repeat expansions, as previously described [17]. Tail and striatum: B6.HdhQ111/+, CAG112; 129.HdhQ111/+, CAG110. (TIF) [file pgen.1003930.s001.tif]

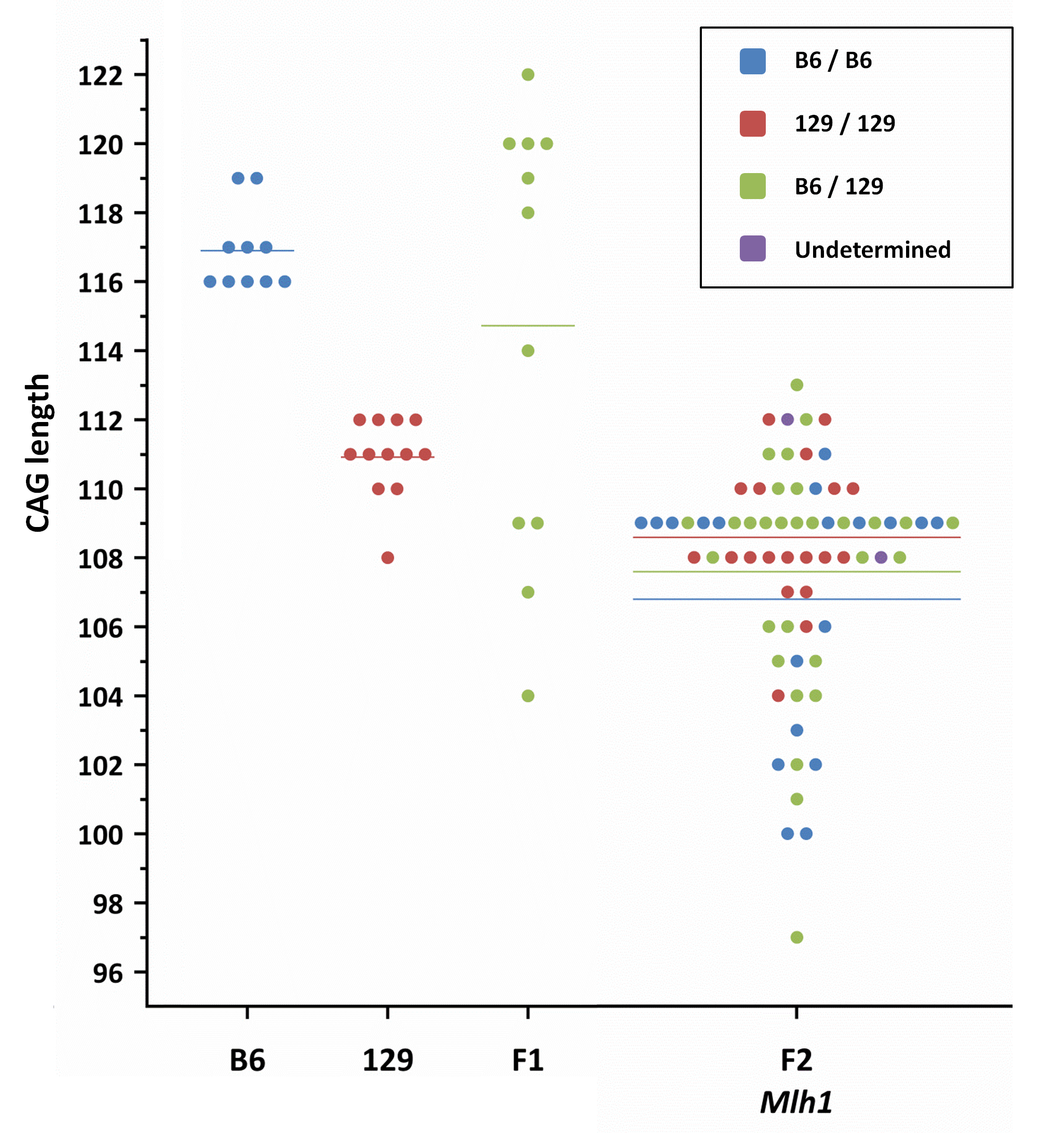

Supplement: Figure S2 — CAG repeat lengths of 10-week-old HdhQ111/+ mice on different genetic backgrounds. Graphical representation of CAG repeat lengths of individual mice used in this study, grouped according to genetic background and color-coded based on genotype. F2 mice are color-coded by Mlh1 genotype. Blue: homozygous for B6 alleles; red: homozygous 129; green: heterozygous B6/129; purple: failed genotype. Constitutive Hdh CAG repeat lengths were determined from tail samples. dbSNP markers located within Mlh1 gene: rs30131926 and rs30174694 (concordant genotypes detected with both markers). B6.HdhQ111/+, n = 10; 129.HdhQ111/+, n = 12; (B6x129).HdhQ111/+ F1, n = 11; (B6x129).HdhQ111/+ F2, n = 69. Horizontal bars represent the mean CAG repeat length of respective group. (TIF) [file pgen.1003930.s002.tif]

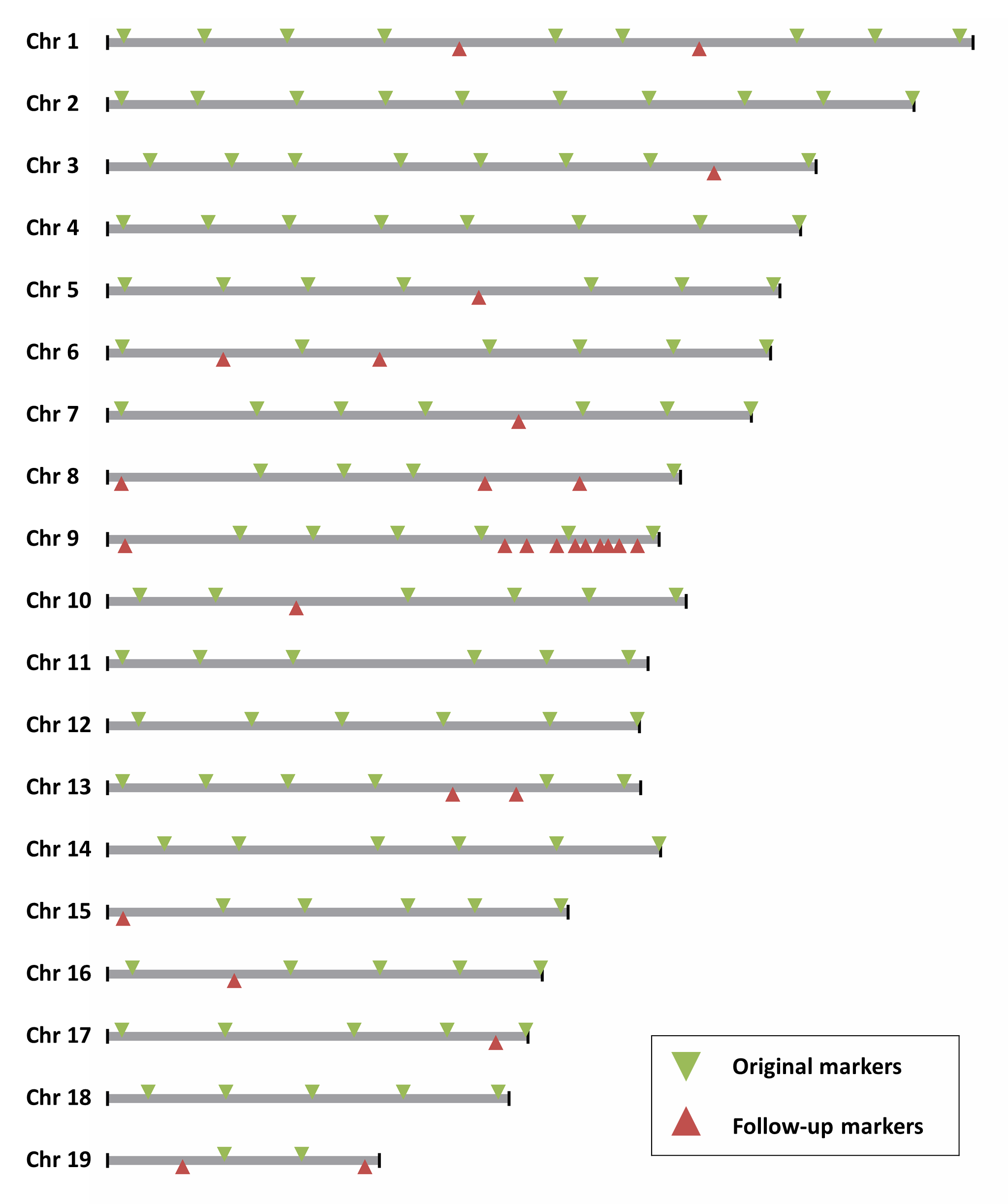

Supplement: Figure S3 — Chromosomal distribution of genetic markers used for QTL analysis. An initial panel of 117 SNPs (green triangles) that distinguish between B6 and 129 strains was used to perform linkage analysis, resulting in the identification of a QTL in chromosome 9 (Figure S4). An additional set of 30 SNPs (red triangles) was subsequently used to enhance resolution at this QTL and improve overall genome coverage, but also to specifically investigate the Mlh1, Msh2 and Msh3 genetic loci (Figure 3). Marker chromosomal positions and dbSNP references can be found in Table S1. (TIF) [file pgen.1003930.s003.tif]

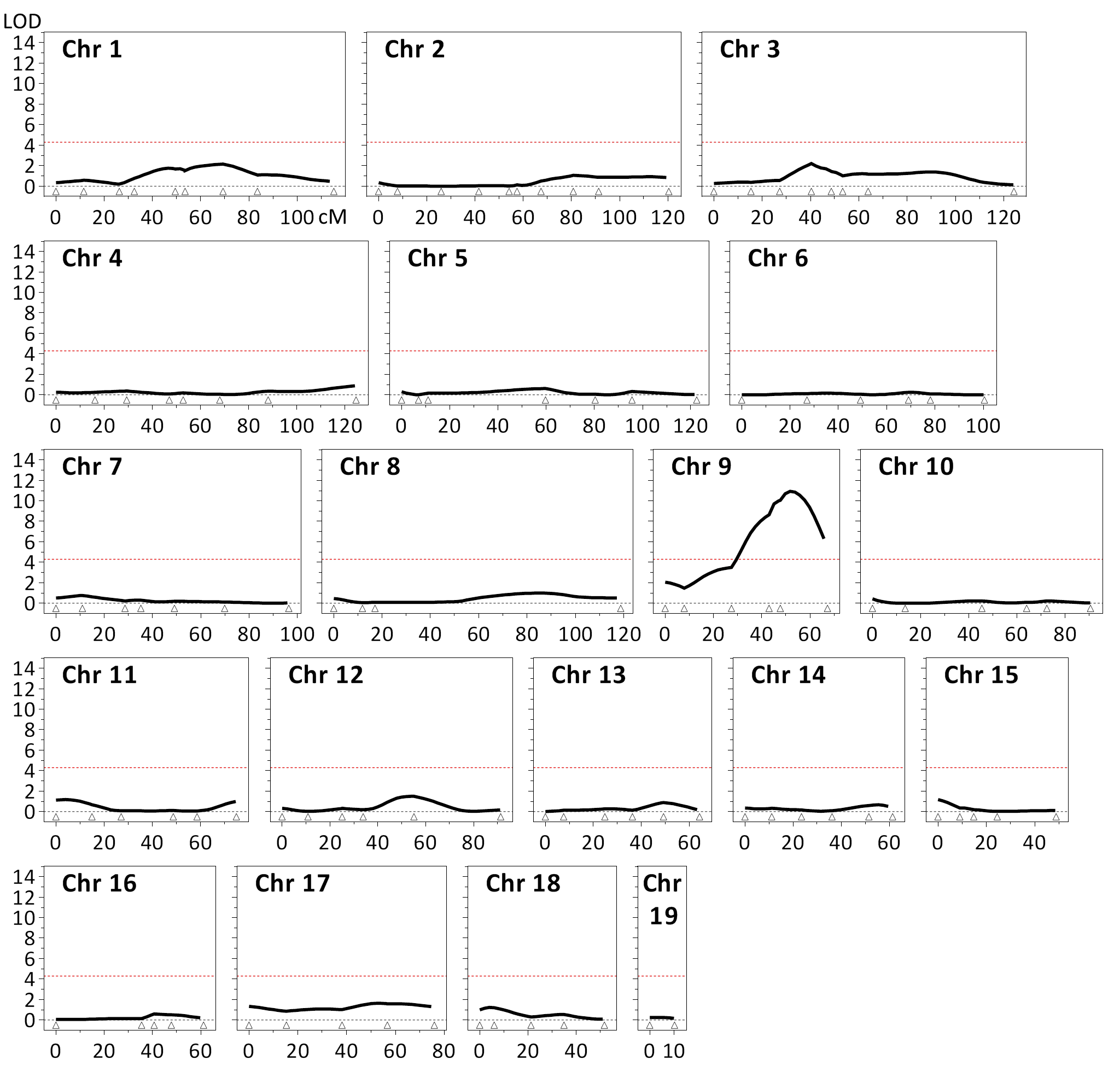

Supplement: Figure S4 — Preliminary mapping of QTL associated with striatal CAG instability. Preliminary linkage analysis in 10-week-old (B6x129).HdhQ111/+ F2 mice (n = 69) identified a single QTL on chromosome 9 with a LOD score of ∼11. The red dashed line represents the threshold (LOD = 4.3) considered for the identification of significant QTLs [85]. The coordinates (cM) of the 117 genetic markers used are represented by open triangles. (TIF) [file pgen.1003930.s004.tif]

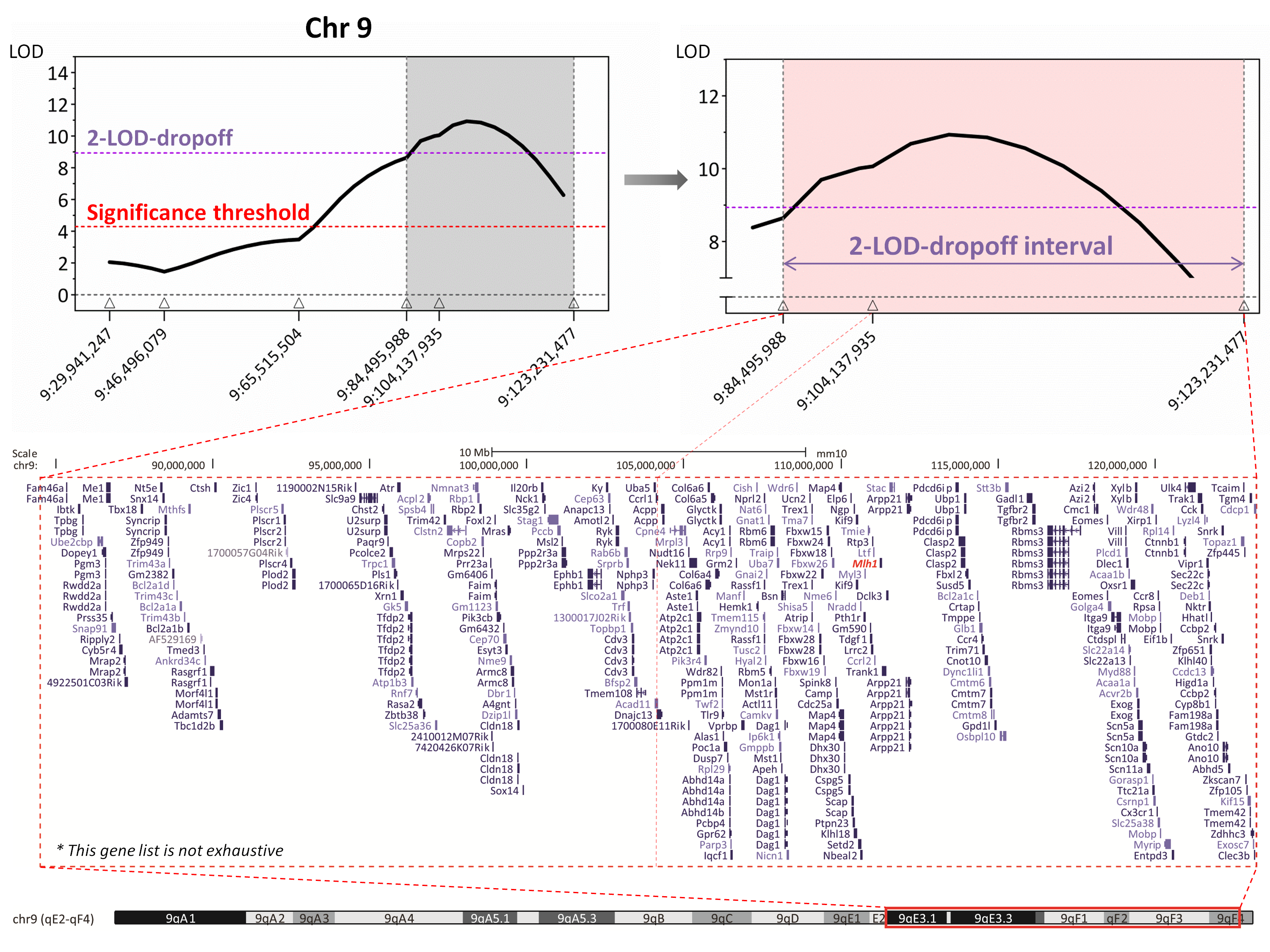

Supplement: Figure S5 — Preliminary mapping of QTL on chromosome 9 implicates numerous genes, including the MMR gene Mlh1. Genome-wide linkage analysis using an initial set of 117 SNPs mapped a single QTL on chromosome 9 strongly linked to striatal CAG instability (Figure S4). A 95% confidence interval was determined by using the 2-LOD-dropoff method [35], [86], implicating a genomic region of approximately 39 Mb (chr9:84,495,988–123,231,477; GRCm38/mm10) that contained numerous genes (∼420), including the MMR gene Mlh1. (TIF) [file pgen.1003930.s005.tif]

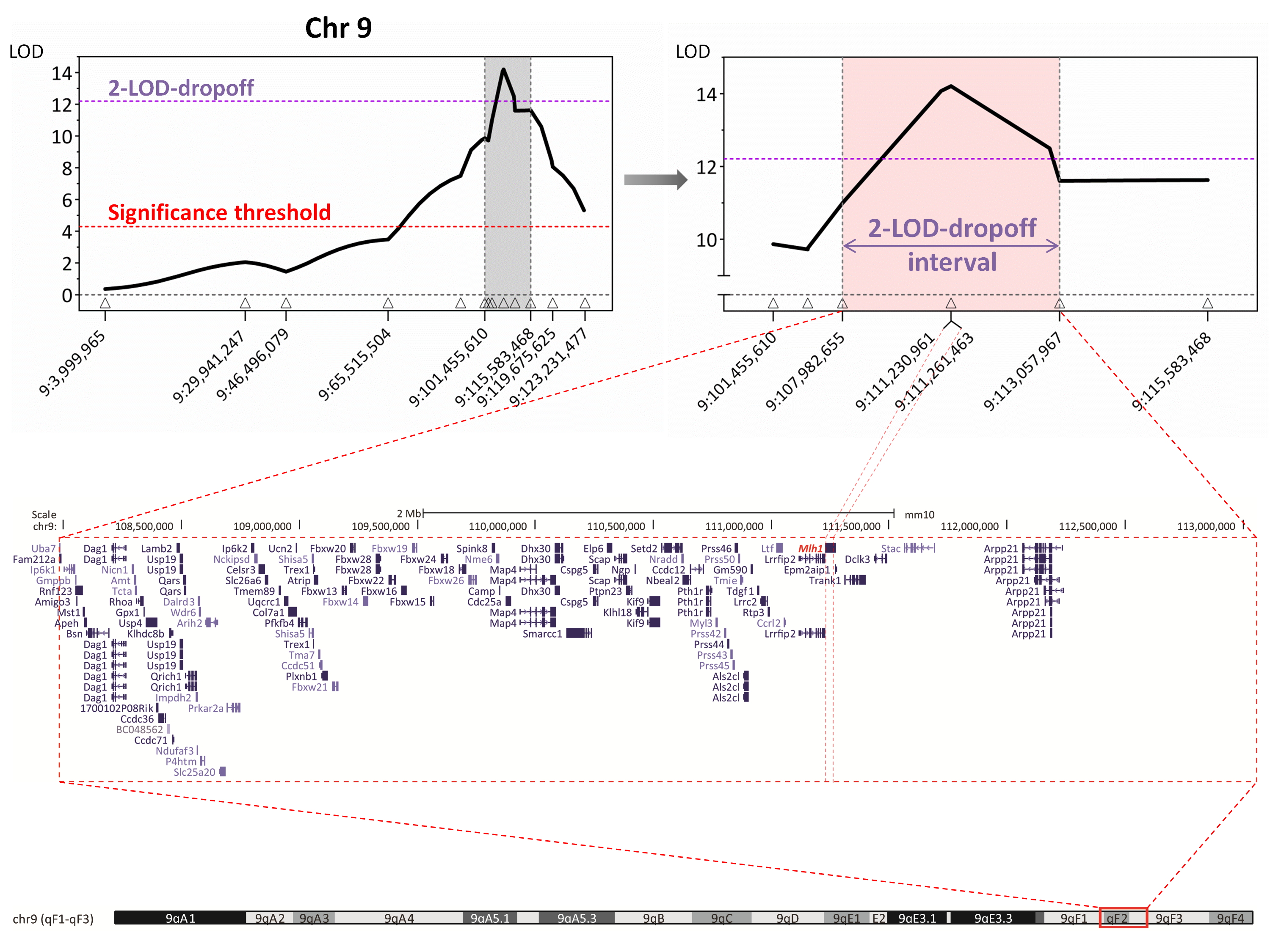

Supplement: Figure S6 — Fine-mapping of chromosome 9 QTL significantly narrowed down the implicated genomic region and number of candidate genes. Follow-up genome-wide linkage analysis with additional genetic markers mapped a single QTL on chromosome 9 strongly linked to striatal CAG instability (Figure 3). A 95% confidence interval was determined by using the 2-LOD-dropoff method [35], [86], narrowing down the implicated region to approximately 5 Mb (chr9:107,982,655–113,057,967; GRCm38/mm10). In addition to Mlh1, the implicated genomic region contains numerous genes (∼100). (TIF) [file pgen.1003930.s006.tif]

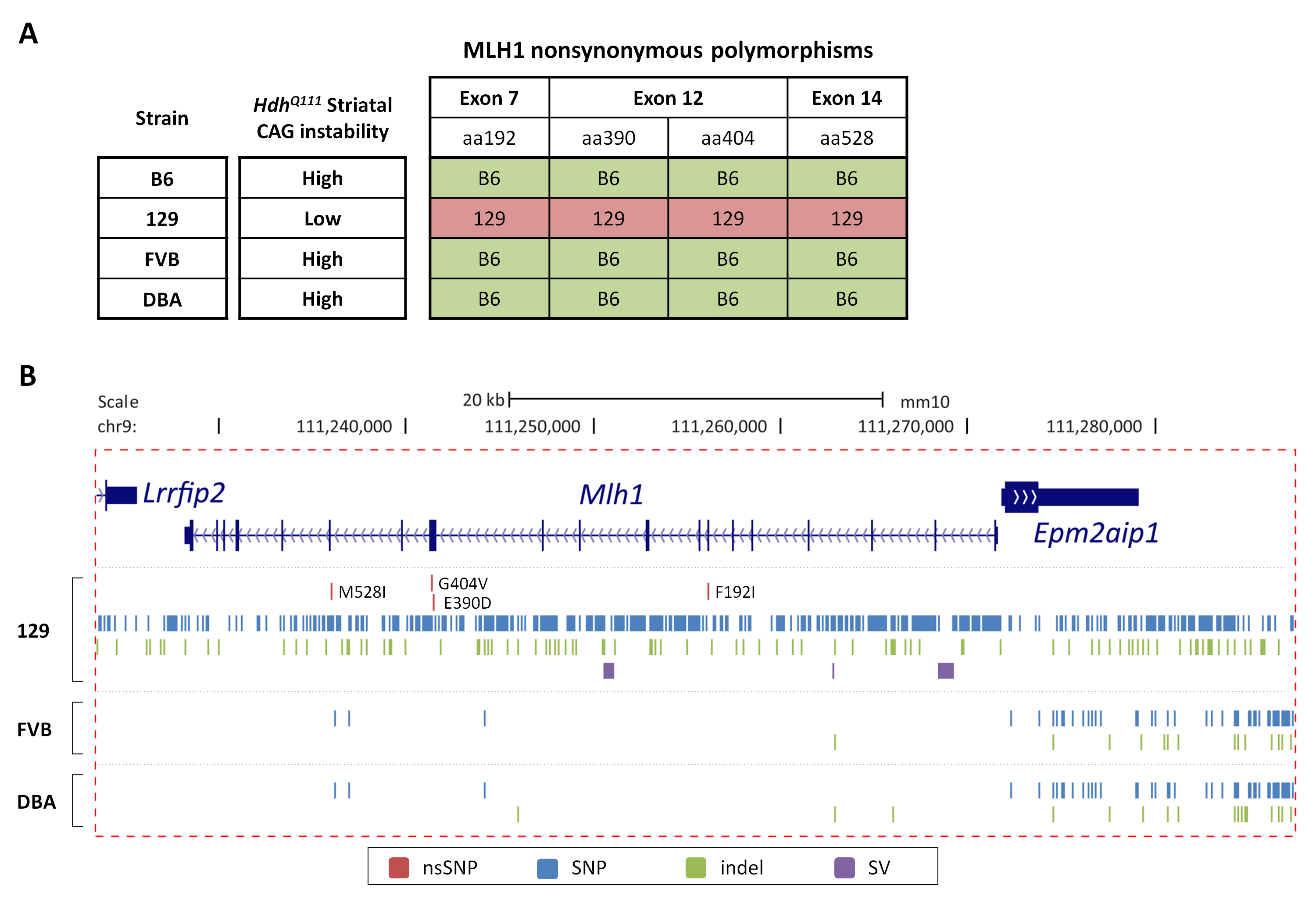

Supplement: Figure S7 — Genetic variation at the Mlh1 locus between different mouse strains. (A) Nonsynonymous polymorphisms identified at the Mlh1 locus in the unstable C57BL/6NCrl, FVB/NCrl and DBA/2J HdhQ111 strains, versus the more stable 129S2/SvPasCrlf HdhQ111 strain. (B) Distribution of polymorphisms identified between C57BL/6NJ, 129S1/SvImJ, FVB/NJ and DBA/2J mouse strains at a 64 kb genomic region encompassing the Mlh1 gene (chr9:111,223,496–111,287,496; GRCm38/mm10) using information from the Mouse Genomes Project [44], [45]. Red, nonsynonymous SNPs (nsSNPs); blue, SNPs; green, short indels; purple, structural variants (SV). (TIF) [file pgen.1003930.s007.tif]

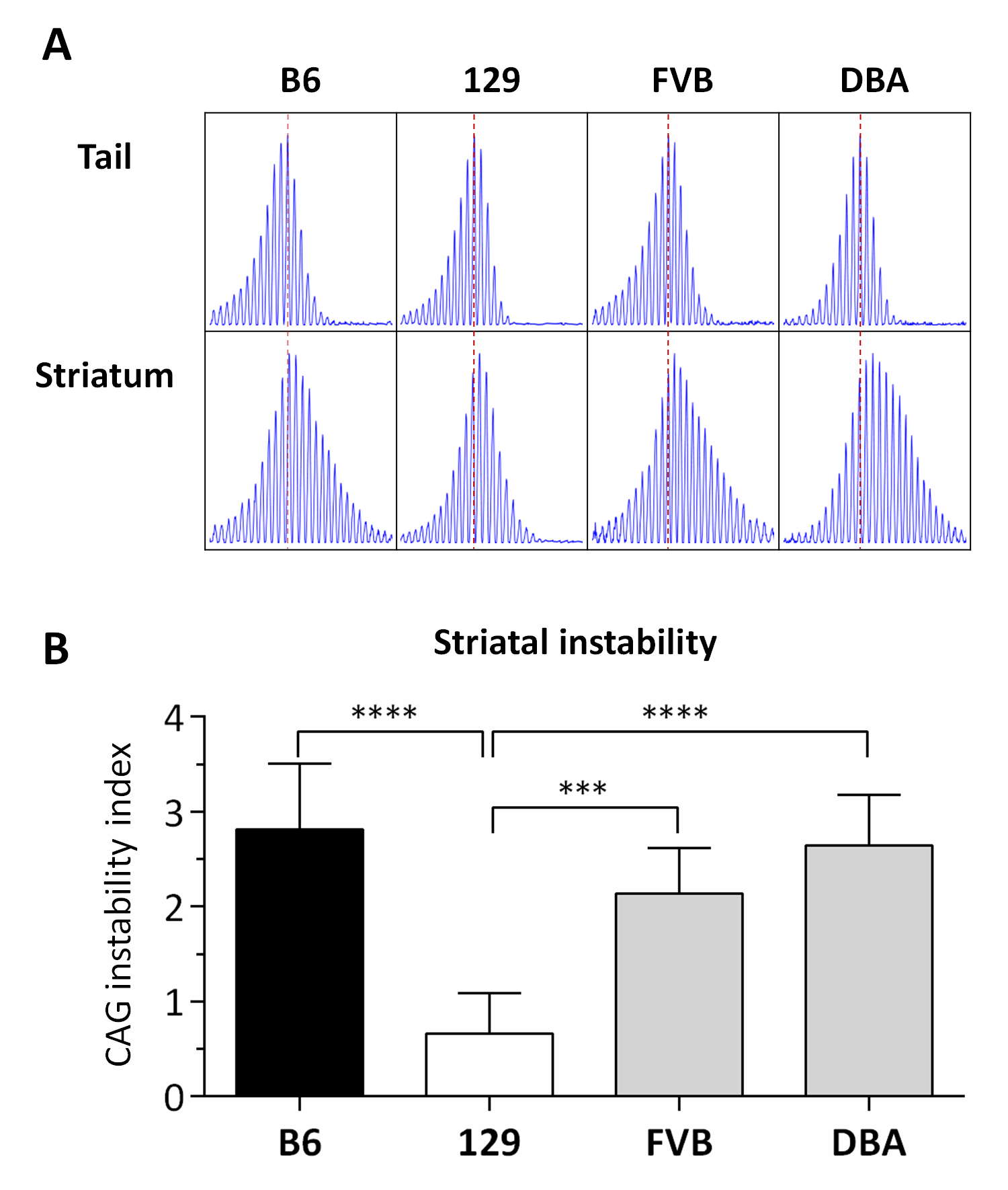

Supplement: Figure S8 — Higher levels of somatic HTT CAG instability in B6, FVB and DBA mice compared to 129. (A) Representative GeneMapper profiles of HTT CAG repeat size distributions in the tail and striatum of 10-week-old C57BL/6NCrl (B6), 129S2/SvPasCrlf (129), FVB/NCrl (FVB) and DBA/2J (DBA) HdhQ111/+ mice, emphasizing the contribution of genetic background to somatic HTT CAG repeat expansion, as previously described [17]. Tail: B6.HdhQ111/+, CAG117; 129.HdhQ111/+, CAG108; FVB.HdhQ111/+, CAG122; DBA.HdhQ111/+, CAG115 (B) Quantification of CAG instability index reveals significantly higher levels of somatic HTT CAG instability in the striatum of B6, FVB and DBA HdhQ111 /+ mice compared to 129.HdhQ111 /+ mice. B6.HdhQ111/+, n = 10, CAG116.9±1.2SD; 129.HdhQ111/+, n = 12, CAG110.9±1.2SD; FVB.HdhQ111/+, n = 3, CAG123.7±2.1SD; DBA.HdhQ111/+, n = 3, CAG115.7±1.2SD; Bar graphs represent mean ±SD; ***, p<0.001; ****, p<0.0001. (TIF) [file pgen.1003930.s008.tif]

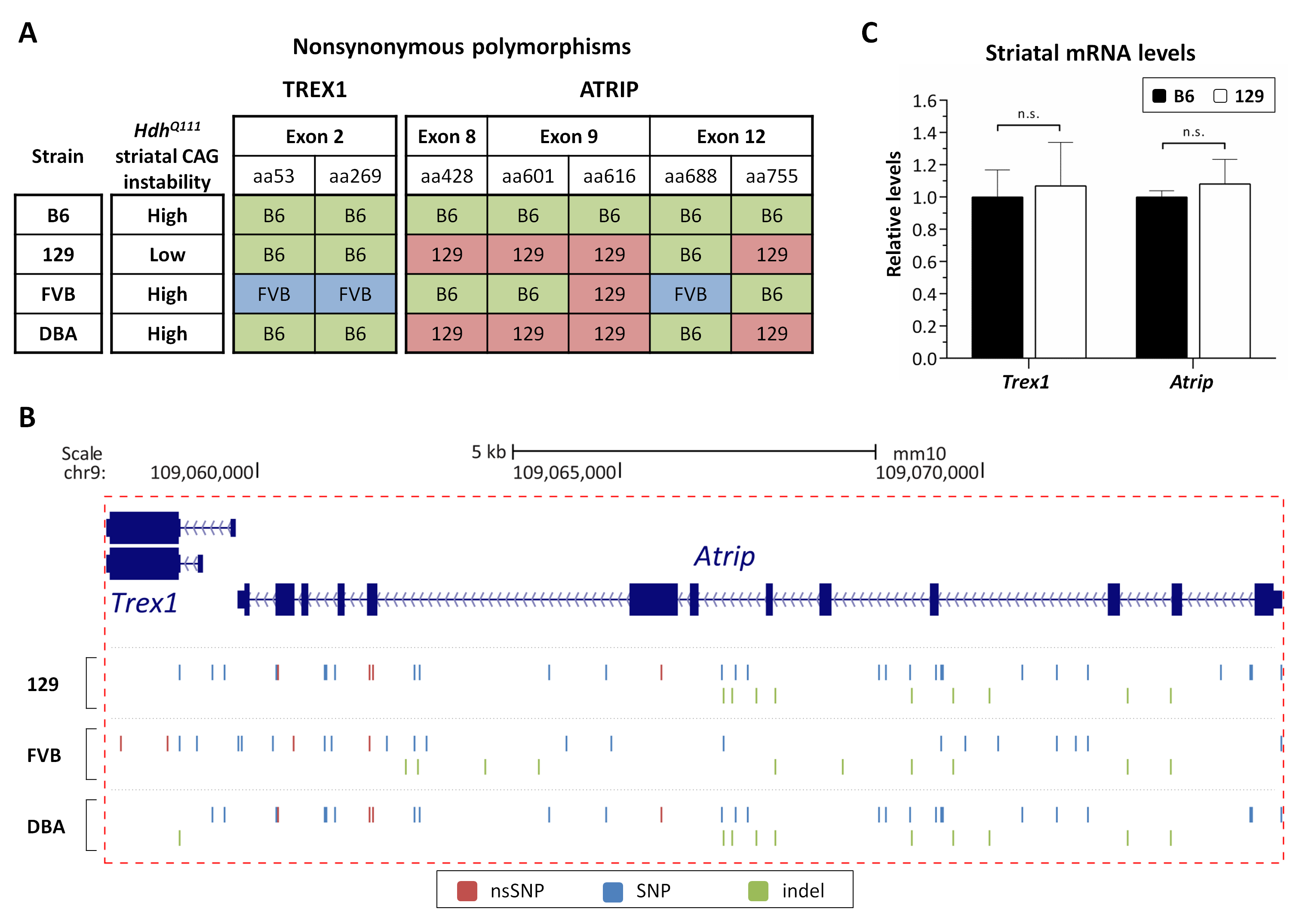

Supplement: Figure S9 — Comparison of Trex1 and Atrip genes in different mouse strains. (A) Nonsynonymous polymorphisms identified at the Trex1 and Atrip locus in the unstable B6, FVB and DBA HdhQ111 strains, versus the more stable 129 HdhQ111 strain. (B) Distribution of polymorphisms identified between B6, 129, FVB and DBA mouse strains at a 16 kb genomic region containing the Trex1 and Atrip genes (chr9:109,057,932–109,074,124; GRCm38/mm10) using information from the Mouse Genomes Project [44], [45]. Red, nonsynonymous SNPs (nsSNPs); blue, SNPs; green, short indels. (C) Quantification of Trex1 and Atrip mRNA levels in the striatum of B6 and 129 10-week-old mice (n = 3) by TaqMan-based qRT-PCR. mRNA levels were determined relative to the housekeeping gene Actb. Bar graphs represent mean ±SD. (TIF) [file pgen.1003930.s009.tif]

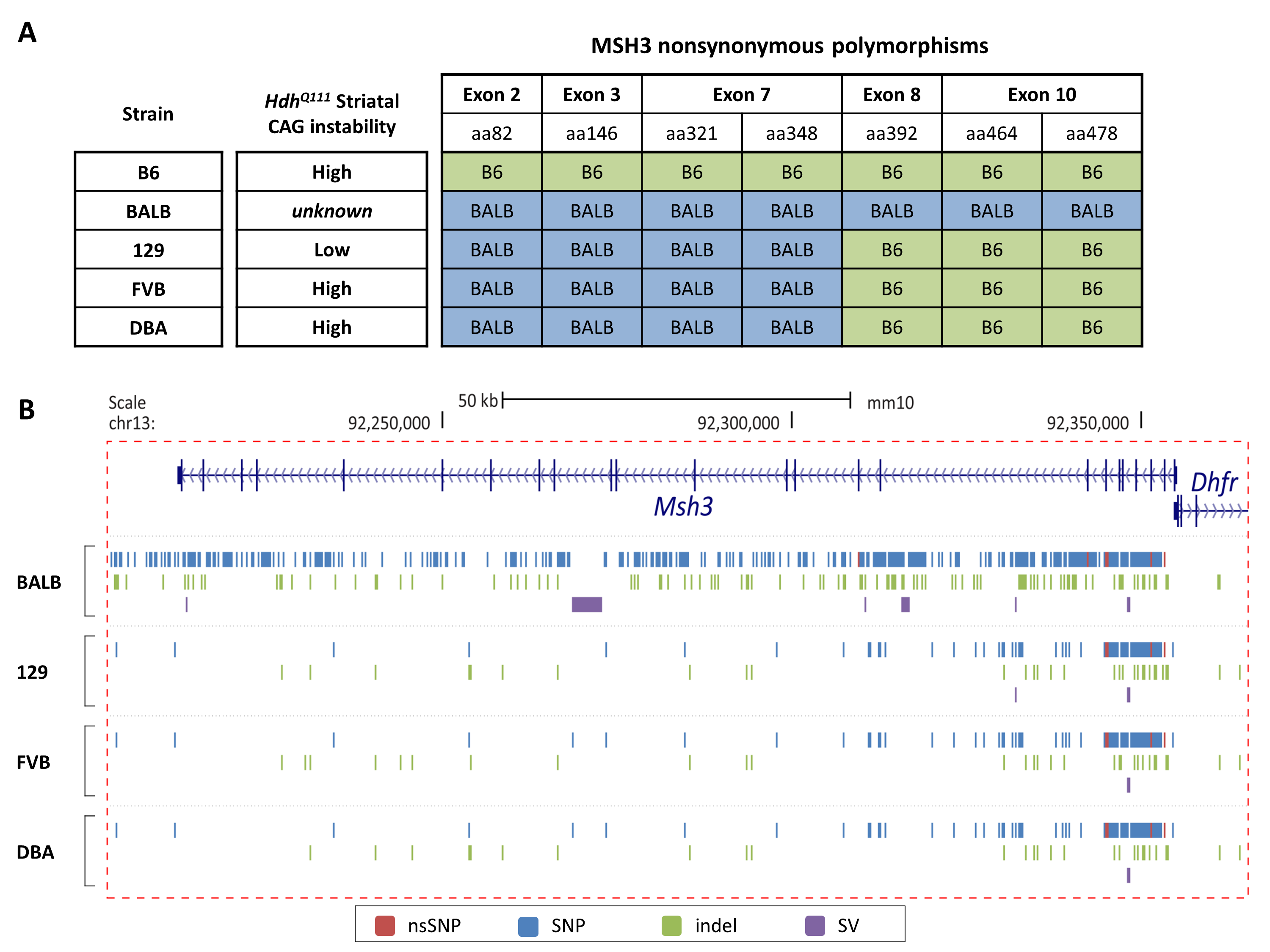

Supplement: Figure S10 — Genetic variation at the Msh3 locus between different mouse strains. (A) Nonsynonymous polymorphisms identified at the Msh3 locus in B6, BALB, 129, FVB and DBA mouse strains. (B) Distribution of genetic polymorphisms identified between C57BL/6NJ, BALB/cJ, 129S1/SvImJ, FVB/NJ and DBA/2J mouse strains across a region encompassing the Msh3 gene (chr13:92,201,881–92,365,003; GRCm38/mm10) using information from the Mouse Genomes Project [44], [45]. Red, nonsynonymous SNPs (nsSNP); blue, SNPs; green, short indels; purple, structural variants (SV). (TIF) [file pgen.1003930.s010.tif]

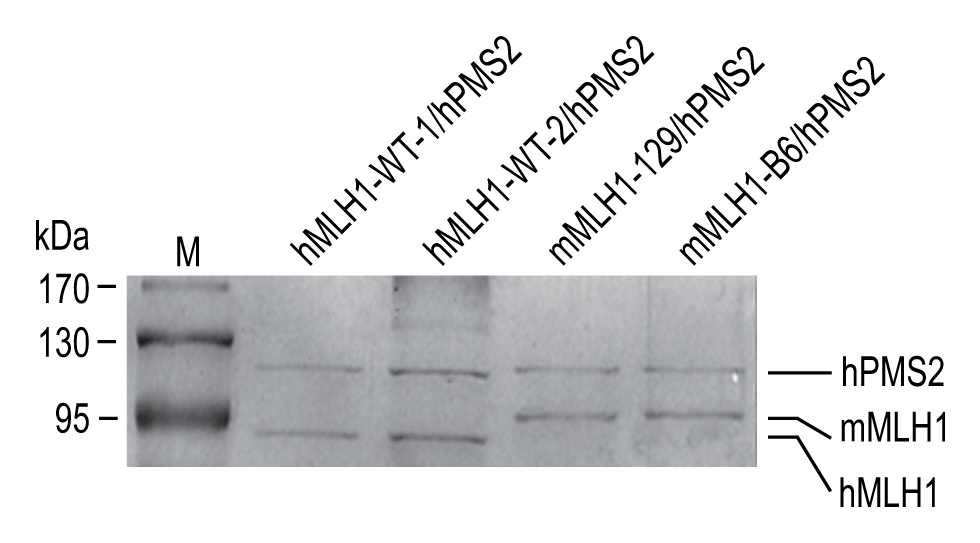

Supplement: Figure S11 — Purified MutLα protein complexes used for cell-free MMR assays. Human and mouse MLH1 proteins from B6 and 129 strains were independently co-expressed with human PMS2 protein in a baculovirus expression system. Purified MutLα complexes were analyzed by polyacrylamide gel electrophoresis and coomassie blue staining. (TIF) [file pgen.1003930.s011.tif]

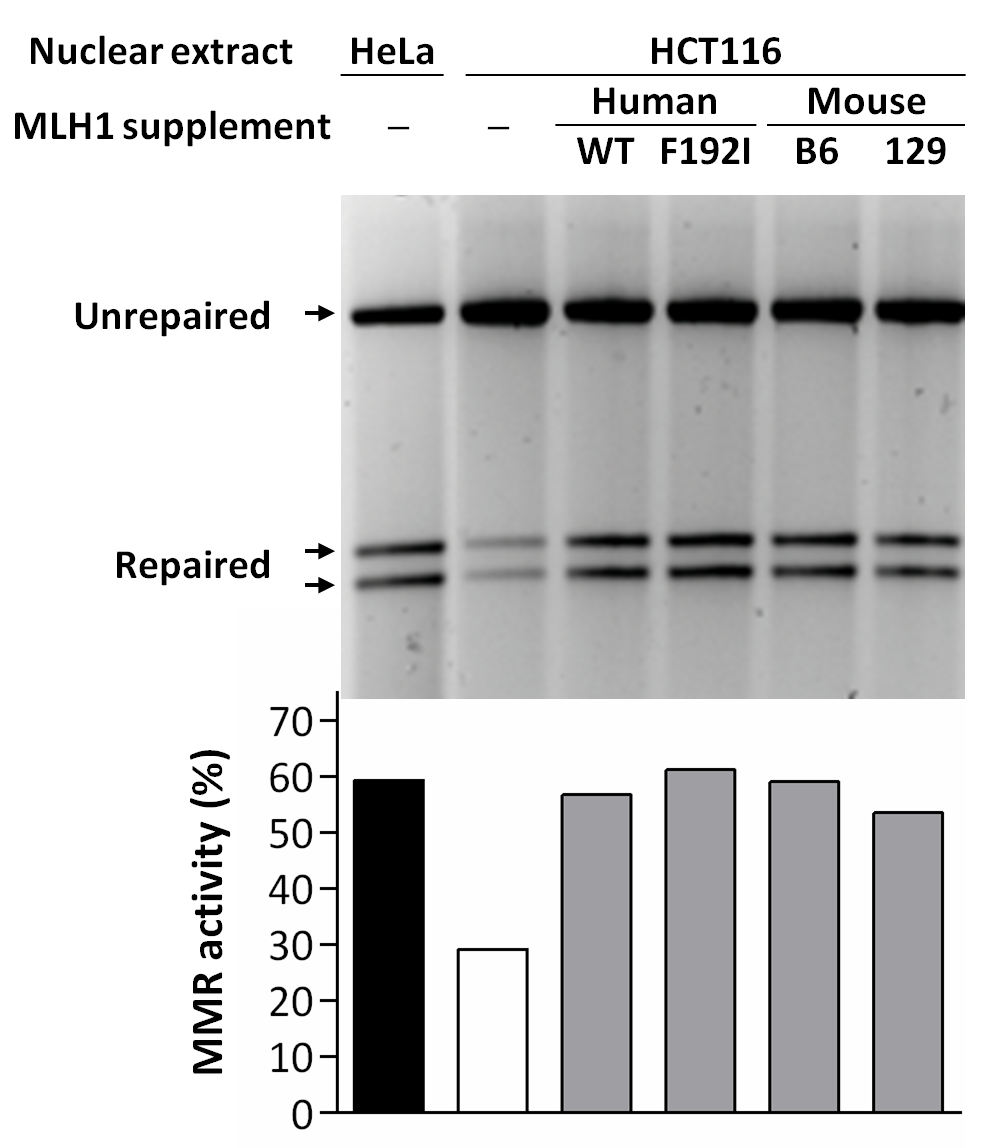

Supplement: Figure S12 — B6 and 129 MLH1 proteins show similar ability to repair single base mismatches in a cell-free MMR assay. Repair of a single base mismatch (G-T) containing 5′ nick using HeLa or HCT116 (MutLα-deficient) nuclear extracts complemented with equal amounts of purified MutLα protein complexes: hMLH1.WT-hPMS2, hMLH1.F192I-hPMS2, mMLH1.B6-hPMS2 or mMLH1.129-hPMS2. Both B6 and 129 MLH1 proteins show ability to repair the mismatch when in a complex with hPMS2, with no overt difference in repair efficiency being observed between the two (lanes 5 and 6). Likewise, introduction of the 129-like F192I mutation in the human MLH1 protein had no discernible effect in mismatch repair efficiency (lane 4). (TIF) [file pgen.1003930.s012.tif]

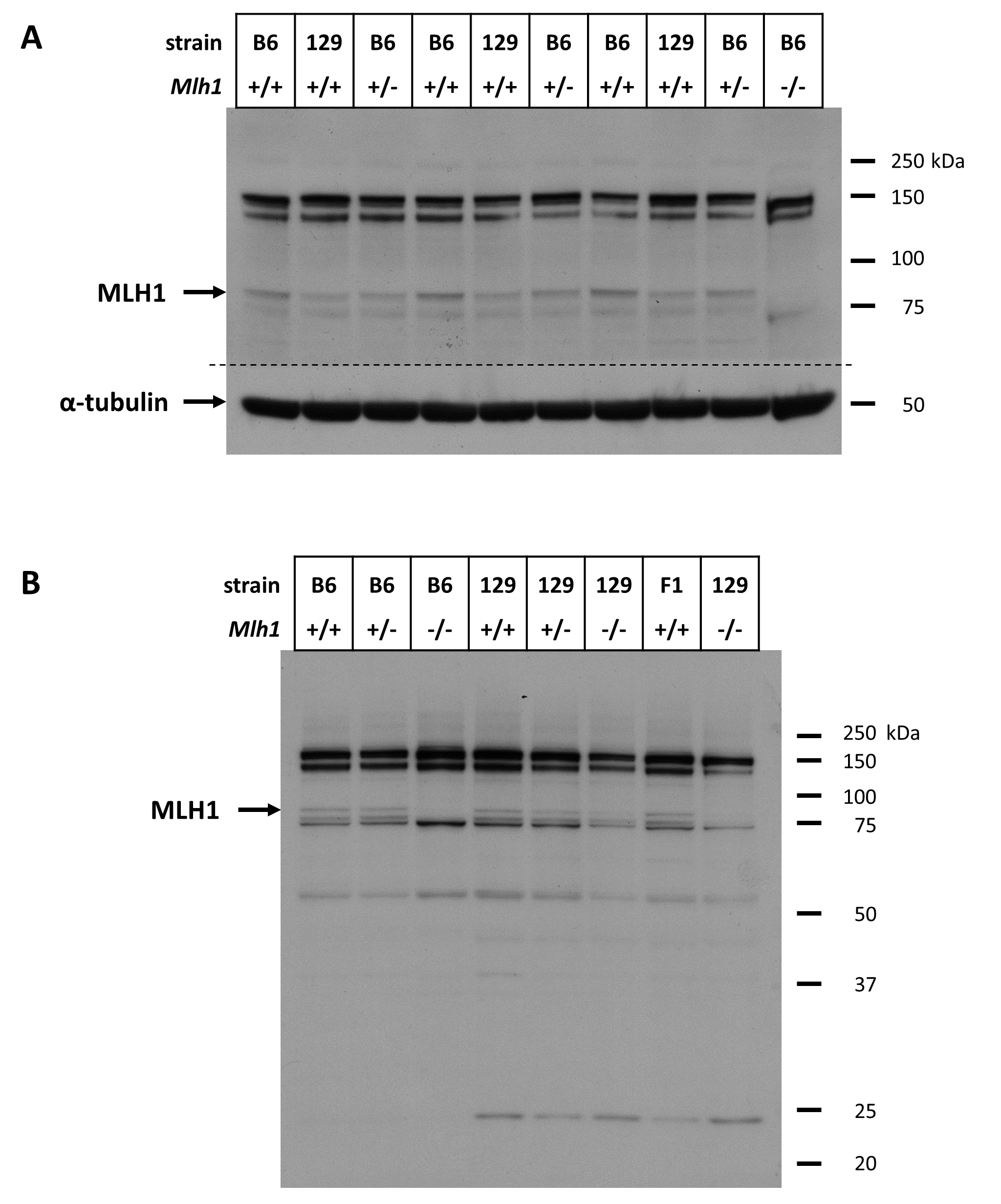

Supplement: Figure S14 — Additional analyses of MLH1 protein by western blot. (A) Representative western blot used for quantification of MLH1 protein levels in the striatum of B6.Mlh1+/+, 129.Mlh1+/+ and B6.Mlh1+/− 10-week-old mice as represented in Figure 9C. The horizontal dashed line represents where the blot was cut (∼60 kDa). The top panel of the blot was probed against MLH1, while the bottom was probed against α-tubulin as loading control. (B) MLH1 western blot of cortex samples from 10-week-old B6 and 129 mice on different Mlh1 genetic backgrounds. (TIF) [file pgen.1003930.s014.tif]
