# Supplementary material for: Mismatch Repair Genes Mlh1 and Mlh3 Modify CAG Instability in Huntington's Disease Mice: Genome-Wide and Candidate Approaches
Source: PLoS Genet. 2013 Oct 31;9(10):e1003930. doi: 10.1371/journal.pgen.1003930 (PMC3814320; doi:10.1371/journal.pgen.1003930)
Supplement: Table S1 — List of genetic markers used for QTL mapping. (PDF) [file pgen.1003930.s015.pdf]

Table S1. List of genetic markers used for QTL mapping.

Marker position	dbSNP	Marker position	dbSNP	Marker position	dbSNP
🟢 1: 3,668,324	rs30610098	🔴 6: 61,462,667	rs3705901	🟢 11: 117,699,263	rs29401815
🟢 1: 21,921,918	rs31130534	🟢 6: 86,309,797	rs30283204	🟢 12: 7,014,383	rs13481285
🟢 1: 40,581,902	rs3677272	🟢 6: 106,716,684	rs30167841	🟢 12: 32,562,930	rs29180162
🟢 1: 62,607,859	rs30661498	🟢 6: 127,777,800	rs29972952	🟢 12: 52,986,606	rs29136254
🔴 1: 79,471,350	rs6260064	🟢 6: 148,811,649	rs30312640	🟢 12: 75,888,429	rs29173065
🟢 1: 101,189,703	rs31967942	🟢 7: 3,131,034	rs31096534	🟢 12: 99,929,915	rs29205196
🟢 1: 116,356,241	rs30523241	🟢 7: 33,737,854	rs31295269	🟢 12: 119,614,608	rs3695641
🔴 1: 133,613,618	rs32624722	🟢 7: 52,768,518	rs32328308	🟢 13: 3,436,259	rs29227900
🟢 1: 155,701,287	rs32026431	🟢 7: 71,780,178	rs31466348	🟢 13: 22,277,704	rs29235380
🟢 1: 173,371,035	rs31066027	🔴 7: 92,859,743	rs3695519	🟢 13: 40,741,808	rs29554010
🟢 1: 192,490,237	rs6277122	🟢 7: 107,335,375	rs31958468	🟢 13: 60,450,061	rs29240914
🟢 2: 3,181,293	rs33618629	🟢 7: 126,417,663	rs3678246	🔴 13: 77,993,752	rs29222817
🟢 2: 20,373,393	rs27120251	🟢 7: 145,321,555	rs31152094	🔴 13: 92,329,978	rs29551174
🟢 2: 42,736,226	rs33122860	🔴 8: 3,130,848	rs32968929	🟢 13: 99,191,767	rs29227075
🟢 2: 62,805,921	rs28002552	🟢 8: 34,563,405	rs32769508	🟢 13: 116,709,204	rs29515886
🟢 2: 80,129,251	rs28324484	🟢 8: 53,390,964	rs32709278	🟢 14: 12,875,703	rs31071846
🟢 2: 102,193,590	rs27375660	🟢 8: 69,066,948	rs3704053	🟢 14: 29,684,270	rs6159786
🟢 2: 122,278,164	rs33269443	🔴 8: 85,228,780	rs33001102	🟢 14: 61,045,685	rs30701326
🟢 2: 143,924,535	rs32853364	🔴 8: 106,640,275	rs33180684	🟢 14: 79,369,412	rs6279914
🟢 2: 161,698,840	rs27333766	🟢 8: 127,928,379	rs33152430	🟢 14: 101,440,731	rs30741554
🟢 2: 181,804,017	rs27670658	🔴 9: 3,999,965	rs31575400	🟢 14: 124,595,261	rs30112843
🟢 3: 9,622,963	rs6387423	🟢 9: 29,941,247	rs30088824	🔴 15: 3,531,273	rs32192585
🟢 3: 28,079,009	rs31636604	🟢 9: 46,496,079	rs30282496	🟢 15: 26,160,100	rs32299863
🟢 3: 42,364,772	rs3665179	🟢 9: 65,515,504	rs29688072	🟢 15: 44,565,754	rs31627972
🟢 3: 66,237,895	rs30760009	🟢 9: 84,495,988	rs30039466	🟢 15: 67,853,911	rs31202853
🟢 3: 84,304,691	rs31311415	🔴 9: 89,738,640	rs33711475	🟢 15: 82,974,731	rs32192579
🟢 3: 103,598,823	rs30303620	🔴 9: 94,703,059	rs3675014	🟢 15: 102,442,365	rs31786273
🟢 3: 122,664,544	rs6381993	🔴 9: 101,455,610	rs29888407	🟢 16: 5,661,761	rs4153209
🔴 3: 136,948,771	rs6228683	🟢 9: 104,137,935	rs30278935	🔴 16: 28,637,123	rs4166709
🟢 3: 158,372,599	rs30263084	🔴 9: 105,635,586	rs29646146	🟢 16: 41,339,150	rs4178232
🟢 4: 3,567,949	rs27720774	🔴 9: 107,982,655	rs30260647	🟢 16: 61,533,650	rs4193306
🟢 4: 22,773,205	rs27745520	🔴 9: 111,230,961	rs30131926	🟢 16: 79,575,938	rs6166418
🟢 4: 41,025,574	rs6232550	🔴 9: 111,261,463	rs30174694	🟢 16: 97,824,456	rs3164033
🟢 4: 61,857,953	rs13477735	🔴 9: 113,057,967	rs30142099	🟢 17: 3,232,805	rs33470793
🟢 4: 81,237,051	rs28106227	🔴 9: 115,583,468	rs3719509	🟢 17: 26,581,579	rs33523502
🟢 4: 106,454,549	rs28187349	🔴 9: 119,675,625	rs30342418	🟢 17: 55,702,582	rs33567983
🟢 4: 133,869,969	rs27594533	🟢 9: 123,231,477	rs29596800	🟢 17: 76,686,931	rs29714724
🟢 4: 156,249,738	rs33891940	🟢 10: 7,281,916	rs29325124	🔴 17: 87,693,244	rs33609112
🟢 5: 3,938,277	rs31193261	🟢 10: 24,408,843	rs29342973	🔴 17: 87,723,010	rs49012398
🟢 5: 26,203,377	rs32285801	🔴 10: 42,660,870	rs29321011	🟢 17: 94,454,832	rs29504100
🟢 5: 45,288,181	rs33309044	🟢 10: 67,884,946	rs6242461	🟢 18: 9,196,202	rs3683892
🟢 5: 66,921,126	rs29730734	🟢 10: 91,928,393	rs29335849	🟢 18: 26,761,256	rs13483264
🔴 5: 83,844,237	rs3712896	🟢 10: 108,757,075	rs29319568	🟢 18: 46,263,795	rs29774134
🟢 5: 109,239,648	rs33582007	🟢 10: 128,435,607	rs29346747	🟢 18: 66,818,290	rs29723033
🟢 5: 129,724,156	rs33689749	🟢 11: 3,350,241	rs26884122	🟢 18: 88,276,319	rs30060354
🟢 5: 150,400,890	rs3165172	🟢 11: 20,922,911	rs3664865	🔴 19: 16,957,814	rs30759036
🟢 6: 3,371,606	rs6392909	🟢 11: 41,923,065	rs29438923	🟢 19: 26,422,340	rs30759358
🔴 6: 26,141,827	rs30812384	🟢 11: 82,876,737	rs29447499	🟢 19: 43,856,492	rs31077420
🟢 6: 43,996,759	rs30316067	🟢 11: 99,212,911	rs29454222	🔴 19: 58,126,053	rs3727025

Marker chromosomal positions in GRCm38/mm10 assembly; 🟢 – Original marker; 🔴 – Follow-up marker
