# Supplementary material for: Mismatch Repair Genes Mlh1 and Mlh3 Modify CAG Instability in Huntington's Disease Mice: Genome-Wide and Candidate Approaches
Source: PLoS Genet. 2013 Oct 31;9(10):e1003930. doi: 10.1371/journal.pgen.1003930 (PMC3814320; doi:10.1371/journal.pgen.1003930)
Supplement: Table S2 — List of primers used. (PDF) [file pgen.1003930.s016.pdf]

Table S2. List of primers used.

Primers used to sequence the *Mlh1* genetic locus:

	Forward primer	Reverse primer
Mlh1 – 5' – 1	5'–CCTATTGGCTGGAGATTTCG–3'	5'–GGCCGCTGAATGACTTCC–3'
Mlh1 – 5' – 2	5'–TAATCCTCTTGGGCGTCATC–3'	5'–CTGATTGGGCAGCATGAAT–3'
Mlh1 – 5' – 3	5'–AACCGCTCGTAGAATTCGTG–3'	5'–ATCTCCAGCCAATAGGAACG–3'
Mlh1 – 5' – 4	5'–ACAGCTGGCTGCGGAGGTTG–3'	5'–CTGGTGGTGGAGCCTGCGG–3'
Mlh1 – 5' – 5	5'–AACAGTTCCAAGTGAAGAAATCCCGAA–3'	5'–ACCAGTGCATGGAGGTGTTGCTG–3'
Mlh1 – 5' – 6	5'–TCTGCAGTTCTTCACTGATCTCTCGAA–3'	5'–TGGACTGACCACGACCCACAC–3'
Mlh1 – 5' – 7	5'–TCTGTACTCATTCCACAGGTCAGTGT–3'	5'–GAAAAGAAATCGAGGCGTTCTTGGTC–3'
Mlh1 – 5' – 8	5'–CAAATCAACTTCTTGGACCACAACGG–3'	5'–CAAATGGTGATCTCCCGCCTACAG–3'
Mlh1 – 5' – 9	5'–CCACGTGTCACACTTTCCAC–3'	5'–ACATTGAGCCAGCCACTGAC–3'
Mlh1 – Exon 1	5'–GGAAGAACTTGAGCGTGAGG–3'	5'–GACTGCAGCAGGTGGGTC–3'
Mlh1 – Exon 2	5'–TCAGAGTAGTTGCAACTGACCAAAT–3'	5'–AGTCTCCAGGCTGGGAAGAG–3'
Mlh1 – Exon 3	5'–AAGATCCAGATTTAAGAAAGCAAAG–3'	5'–TTGCCTCTGTTGAGAAATGAG–3'
Mlh1 – Exon 4	5'–CGATAACCTTTGCCTTGATG–3'	5'–AAATCAGTATCCTAGCGGGG–3'
Mlh1 – Exon 5	5'–GCAGACATAGGCCACTAGGC–3'	5'–TCAGAGCGCATCTCAGTTTTAC–3'
Mlh1 – Exon 6	5'–TTTGGTAGACTGTGCGATGC–3'	5'–ATTTACAAGCACCAGGGCAC–3'
Mlh1 – Exon 7	5'–TCGCTGAATTTATTGTGTGTTTG–3'	5'–TGCAGGCAAAAGTTTACACAC–3'
Mlh1 – Exon 8	5'–AAAACATTCTTGGGGCTTTC–3'	5'–AACTGGGGTGACAATCAAGG–3'
Mlh1 – Exon 9	5'–TTGTGGGGTAAAGATGGTTTATG–3'	5'–TAATCAGGCCCGACTGTGAC–3'
Mlh1 – Exon 10	5'–ATGCAGCTGTGACCTCATC–3'	5'–TTTCAGAGCAGTGACAAGGC–3'
Mlh1 – Exon 11	5'–CCTTTTCCATGTTGTCTAGTCG–3'	5'–GGAACACATTTCAAAGCAGAG–3'
Mlh1 – Exon 12	5'–CACTGCTGTTCTGTTTTGGG–3'	5'–TAAACAAGGCCACCACACTG–3'
Mlh1 – Exon 13	5'–TTTCTCCAGAGTATTTAGCTGGG–3'	5'–CCTGAGTGCTTTCTGCATTTG–3'
Mlh1 – Exon 14	5'–TGTGTGGGACTGGAGGTATG–3'	5'–CTCCTGCACTGGTGCCTATC–3'
Mlh1 – Exon 15	5'–AGAGGACCTCCTTAGCCAGG–3'	5'–AACCACACGACGGCTCAC–3'
Mlh1 – Exon 16	5'–ACTGAGCTTGCTGAGACAGG–3'	5'–AAAGACATCCAGATGGCCC–3'
Mlh1 – Exon 17	5'–TTGGAGAAGCACCAGAGACA–3'	5'–TCTTTTCCATTCCAAATGAAGG–3'
Mlh1 – Exon 18	5'–GCCAGGTCTTAGTGCCAGAA–3'	5'–CCCATACCCTAAAGTCATGAGA–3'
Mlh1 – Exon 19	5'–ACCACTGAGACCTTTCCAGG–3'	5'–TACCATACACTTCGCCTTGG–3'
Mlh1 – 3' – 1	5'–CCGCTCACACCTCCTACCTCCG–3'	5'–AGAGTGTGTGTAGAAATGCCTCCTCA–3'
Mlh1 – 3' – 2	5'–GCTCCAGGGTTTCCAGTGCTCACTA–3'	5'–GTGCCCAACCTTGGGATTTGTTTTG–3'
Mlh1 – 3' – 3	5'–TCTGATGGTGGTGGTCTGGAAAGC–3'	5'–TGGAAGTGGTTCCTGCTGCCAA–3'
Mlh1 – 3' – 4	5'–GTAAGTCAGTCTTGTCAGTTGATTGGA–3'	5'–ACTCCAAACTTCTACATCCCTCTTGTA–3'
Mlh1 – 3' – 5	5'–GCGAACCCTGCCTAAAAGTGACAC–3'	5'–TCACCTTTGCCATTGCCCAGTAC–3'
Mlh1 – 3' – 6	5'–TTGAGGTTGATAGTGACAATTCAGA–3'	5'–ACAGCCATGAAGTGGGCTAA–3'

Primers used for cloning the *Mlh1* cDNA:

Mlh1 cDNA – NcoI – F	5'–ATTCAAGAATTCCCATGGCGTTTGTAGCAGGAGTTATTC–3'
Mlh1 cDNA – XhoI – R	5'–ATTCAAGAATTCCTCGAGTTAACACCGCTCAAAGACTTTGTATAG–3'

Primers used to sequence the *Mlh1* cDNA:

Mlh1 cDNA – 1 – R	5'–TTGGGCAGTGTTCTGACATC–3'
Mlh1 cDNA – 2 – R	5'–GCTTACAGGCTGCAGAAAGG–3'
Mlh1 cDNA – 3 – F	5'–TTTCACCCAGACCTTGCTTC–3'
Mlh1 cDNA – 4 – F	5'–CCTCCTCAACACTACCAAGCTC–3'

Primers used to generate *Mlh1*–luciferase reporter constructs:

5’ constructs (2.5 kb)	
F – KpnI	5'–ATTCAAGAATTCGGTACCGCTTCAGTCAGTCCCATGTTGCCAA–3'
R (B6) – NheI	5'–ATTCAAGAATTCGCTAGCATTGGCGCCCGAGTTCCCAG–3'
R (129) – NheI	5'–ATTCAAGAATTCGCTAGCATTGGCGCCAGAGTTGCCCG–3'
3’ constructs (1.7 kb)	
F – NheI	5'–ATTCAAGAATTCGCTAGCGTATACAATCATAGCCACCGTAGAGACTG–3'
R – BamHI	5'–ATTCAAGAATTCGGATCCTCACCTTTGCCATTGCCCAGTAC–3'
3’ constructs (1.3 kb)	
F – NheI	5'–ATTCAAGAATTCGCTAGCGTATACAATCATAGCCACCGTAGAGACTG–3'
R – BamHI	5'–ATTCAAGAATTCGGATCCACTCCAAACTTCTACATCCCTCTTGTA–3'
3’ constructs (0.6 kb)	
F – NheI	5'–ATTCAAGAATTCGCTAGCGTATACAATCATAGCCACCGTAGAGACTG–3'
R – BamHI	5'–ATTCAAGAATTCGGATCCGTGCCCAACCTTGGGATTTGTTTTG–3'
3’ constructs (0.2 kb)	
F – NheI	5'–ATTCAAGAATTCGCTAGCGTATACAATCATAGCCACCGTAGAGACTG–3'
R – BamHI	5'–ATTCAAGAATTCGGATCCACCAATACACGTGAGTTTATTTGCAGC–3'
3’ “swap” constructs (1.7 kb)	
F – NheI	5'–ATTCAAGAATTCGCTAGCGTATACAATCATAGCCACCGTAGAGACTG–3'
R – NheI	5'–ATTCAAGAATTCGCTAGCTATCACCTTTGCCATTGCCCAGTAC–3'
